# Hemodynamic functional connectivity optimization of frequency EEG microstates enables attention LSTM framework to classify distinct temporal cortical communications of different cognitive tasks

**DOI:** 10.1186/s40708-022-00173-5

**Published:** 2022-10-11

**Authors:** Swati Agrawal, Vijayakumar Chinnadurai, Rinku Sharma

**Affiliations:** 1 Institute of Nuclear Medicine and Allied Sciences, Lucknow Road, Timarpur, Delhi, 110054 India; 2 Delhi Technological University, Shahbad Daulatpur, Main Bawana Road, Delhi, 110042 India

**Keywords:** Attention-based deep learning, Frequency-microstates, Hemodynamic functional connectivity, EEG-informed fMRI. Multifrequency coupling, Target detection

## Abstract

Temporal analysis of global cortical communication of cognitive tasks in coarse EEG information is still challenging due to the underlying complex neural mechanisms. This study proposes an attention-based time-series deep learning framework that processes fMRI functional connectivity optimized quasi-stable frequency microstates for classifying distinct temporal cortical communications of the cognitive task. Seventy volunteers were subjected to visual target detection tasks, and their electroencephalogram (EEG) and functional MRI (fMRI) were acquired simultaneously. At first, the acquired EEG information was preprocessed and bandpass to delta, theta, alpha, beta, and gamma bands and then subjected to quasi-stable frequency-microstate estimation. Subsequently, time-series elicitation of each frequency microstates is optimized with graph theory measures of simultaneously eliciting fMRI functional connectivity between frontal, parietal, and temporal cortices. The distinct neural mechanisms associated with each optimized frequency-microstate were analyzed using microstate-informed fMRI. Finally, these optimized, quasi-stable frequency microstates were employed to train and validate the attention-based Long Short-Term Memory (LSTM) time-series architecture for classifying distinct temporal cortical communications of the target from other cognitive tasks. The temporal, sliding input sampling windows were chosen between 180 to 750 ms/segment based on the stability of transition probabilities of the optimized microstates. The results revealed 12 distinct frequency microstates capable of deciphering target detections' temporal cortical communications from other task engagements. Particularly, fMRI functional connectivity measures of target engagement were observed significantly correlated with the right-diagonal delta (*r* = 0.31), anterior–posterior theta (*r* = 0.35), left–right theta (*r* = − 0.32), alpha (*r* = − 0.31) microstates. Further, neuro-vascular information of microstate-informed fMRI analysis revealed the association of delta/theta and alpha/beta microstates with cortical communications and local neural processing, respectively. The classification accuracies of the attention-based LSTM were higher than the traditional LSTM architectures, particularly the frameworks that sampled the EEG data with a temporal width of 300 ms/segment. In conclusion, the study demonstrates reliable temporal classifications of global cortical communication of distinct tasks using an attention-based LSTM utilizing fMRI functional connectivity optimized quasi-stable frequency microstates.

**Supplementary Information:**

The online version contains supplementary material available at 10.1186/s40708-022-00173-5.

## Introduction

### Decoding distant cortical communications from cortical EEG

The human brain dynamically engages distinct neural populations between distant brain regions, and their spatiotemporal oscillations often modulate systematically with behavioral and cognitive tasks. The synchrony or lack thereof between remote brain regions brings effective global brain communication, functional connectivity for information processing. Many researchers [25, 31, 35] employed simultaneous EEG information acquired with fMRI imaging to understand these functional connectivities, neural origins, and correlated brain states. These researches revealed that the elicitation of local neural systems is observed as high-frequency dynamics in EEG cortical oscillations. Cortical high-frequency gamma oscillation mainly explains local high-level neural information and positively correlates with the fMRI BOLD strength. Further, the distant cortical and long-range coordination emerges as the lower EEG cortical frequency oscillation [9]. The alpha and beta power modulate the BOLD response's latency and strength to gamma power changes [34]. Many researchers [2, 22, 40] have recently observed multi-frequency cortical EEG interaction explaining hemodynamic task elicitation better than single EEG power information. JC Pang et al. [44] revealed that the inverse correlation of alpha and BOLD originates from high- and low-frequency components of the same underlying neural engagement caused by modulation in corticothalamic and intra-thalamic feedback. Despite these clear insights of distinct global and local neural processing associated with every task engagement, decoding their temporal dynamics computationally from spatially coarse-grained cortical times-series EEG information is still challenging.

### Microstates and global cortical communication

Microstates are the cluster centers of EEG information and are unique in every cognitive task engagement in healthy and disease populations. Every microstate topography is associated with a "quasi-stable" functional state [16, 36], explaining the brain's specific neural interaction. These quasi-stable patterns span around 100 ms and are the most robust approach to bringing distant functional communication in cortical EEG information. Many researchers [24, 64] observed that the time course of microstate metrics, when correlated with the fMRI BOLD signal, reveals functional networks similar to the resting-state networks. Further, the microstate dynamics are observed to measure transitions between global cortical communications characterized by specific local neural alpha inhibitions [12, 23, 38, 56]. The traditional microstate estimation employs the EEG frequency range [30, 45] of 2–20 Hz. Hence, their time-series dynamics are primarily influenced by the cortical alpha inhibitory/excitatory modulations [37]. However, the cognitive task engagement's distant and local cortical communication manifests through other EEG rhythms [18, 49]. Thus, the task-induced modulation of cortical communication and associated local neural engagement is manifested as a combination of different frequencies [4]. More recently, the beta-band and the coverage feature of the EEG microstate analysis have been revealed as the essential features for classifying epilepsy and PNES patients with reasonably high accuracy and precision [3].

### Deep learning approaches for temporal EEG analysis

The EEG time-series information is higher-dimensional, and the cognitive information is spread across its timelines. Hence, the feature information derived from a single time point of EEG time-series data is inadequate to explain any cognitive process. Thus, Recurrent Neural networks (RNN) perform better in extracting sequential information embedded in higher dimensional EEG time-series information. However, the traditional RNN system suffers in learning long-term dynamics due to vanishing/exploding gradient problems. Long Short-Term Memory (LSTM) architecture addresses this exploding gradient obstacle by learning both long- and short-term dependencies. Recently, the attention mechanism has been introduced [55] to improve the performance of deep learning models; it highlights the more informative feature and subsequently gives higher weights to the corresponding original feature sequence. It has been embedded with the LSTM architecture in several EEG studies [21, 28, 48, 61, 62, 66, 67] by effectively selecting the feature information and observed with significantly improved efficiency and performance accuracy of deep learning systems.

### Present study

The present study proposes an attention-based LSTM computational model that employs optimized frequency microstates to decipher the distant cortical communication of visual target detection tasks. The temporal dynamics of the frequency microstate metrics are correlated with fMRI hemodynamic functional connectivity measures to optimize the cortical EEG quasi-stable frequency patterns with the task's local/global brain communication. The hemodynamic functional connectivity is assessed by employing the graph-theoretical analysis on simultaneously acquired fMRI information. The significantly correlated frequency microstates are further subjected to the robust correlation analysis to understand their multi-frequency coupling elicited during intercortical interaction during the task engagement. The local and global neural mechanisms underlying these frequency quasi-stable microstates were further estimated through EEG-informed fMRI analysis. Finally, a hybrid deep learning framework consisting of LSTM with an attention mechanism is employed to classify the target detection task engagement from the temporal dynamics of these optimized frequency microstate quasi-stable patterns. The performance metrics such as precision, accuracy, and recall are estimated for all deep learning architectures and validated using a tenfold cross-validation approach.

## Methods and materials

### Participants and task design

Seventy healthy right-handed volunteers (30 males and 40 females; mean age: 23 years; age range, 20–32 years) were selected from the academic environment. All participants gave written informed consent and did not have psychiatric or neurological disorders or medication. The experiment was conducted following the World Medical Association (Declaration of Helsinki), and the local ethical committee approved all measurements. The vision of participants was corrected using MR-compatible lenses whenever required.

**Fig. 1:**
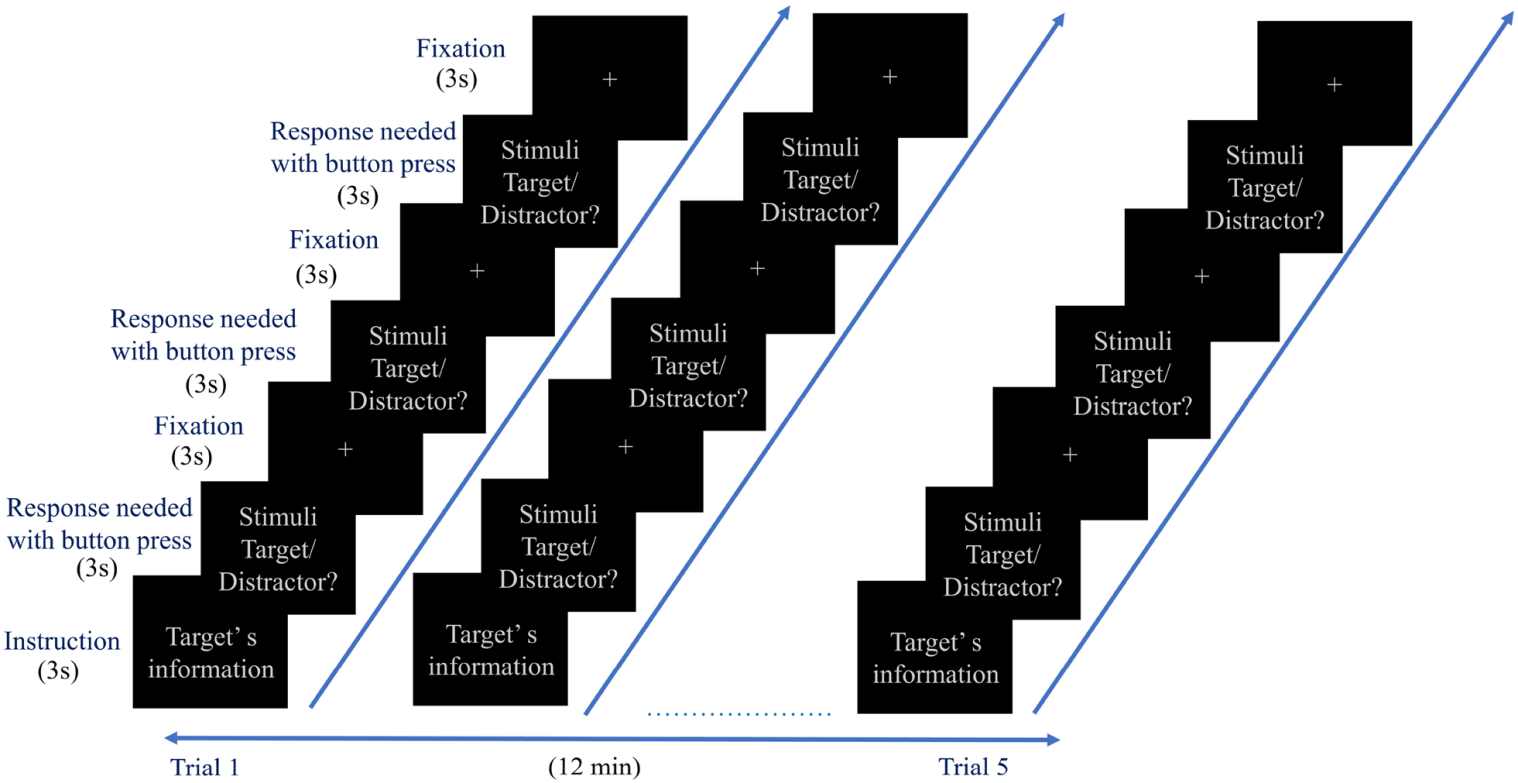
Schematic of task paradigm design

These participants underwent the task paradigm (Fig. 1) designed to perform three tasks. They are target detection, distractor detection, and fixation identification. For this purpose, the multiple distinct shapes (squares, circles, stars, and triangles) with primary colors (experimental conditions, stimuli) were shown at the beginning (called 'Targets stimulus'). Then volunteers were sequentially shown a single stimulus, one at a time, and asked whether it was part of earlier displayed target stimuli collections. The volunteer must click the right thumb button whenever he identifies the target stimulus. Only 30 percent of stimuli were selected from earlier displayed stimuli, the targets, and randomly distributed among the "Distractor Stimulus". The correct identification of the target stimulus invokes the elicitation of distinct neural mechanisms compared to the identification of distractors. Each sequential stimulus was presented for the duration of 3000 ms. The paradigm consisted of five trials. Each trial started with a different target stimuli collection, and 105 single stimuli were subsequently presented for target/distractor identification. Among 105 stimuli, only 32 were target stimuli and presented randomly. A fixation stimulus followed each stimulus; a single black-colored cross in the slide's center was presented for 3 s. These task stimuli are projected onto MR-compatible lenses fitted on the head coil inside MRI.

### Simultaneous EEG–fMRI acquisition and preprocessing

In the present study, simultaneous EEG–fMRI data acquisition is carried out using a 3T Siemens Magnetom Skyra scanner (Siemens, Erlangen) and MR-compatible 32-channel Brain Amp system with an EEG cap. EEG signals are recorded at a sampling rate of 5 kHz, and the impedance of all scalp electrodes is maintained below five kOhms throughout the recording. The fMRI were acquired using Eco Planar Imaging (EPI) sequence with TR = 3000 ms; TE = 36 ms; voxel size = 3.6*3.6*3.0 mm; matrix = 64*64; FoV read = 230 mm; flip angle = 90°, 36 axial slices. The axial slices are acquired parallel to the anterior–posterior (AC–PC) line in an interleaving manner with a slice thickness of 3.0 mm. The high-resolution structural images of the brain are acquired using T1 MPRAGE sequence with parameters, voxel size = 1.0*1.0*1.0 mm; TR = 2000 ms; flip angle = 90°; FoV = 240 mm; matrix = 512*512; slice thickness = 1.0 mm, 160 axial slices. The axial slices are acquired parallel to the anterior–posterior (AC–PC) line in an interleaving manner with a slice thickness of 5.0 mm.

EEG data acquired simultaneously inside the MRI scanner are initially subjected to the FMRIB plugin of EEGLAB to remove the MRI-related artifacts such as gradient switching and cardio ballistic artifacts. The gradient artifact removal process employed FASTR (FMRI Artifact Slice Template Removal) tool [20, 42]. It aligned all the artifact slices to correct any slight jitter in the exact location of the slice-timing events, interpolating and shifting each artifact until the correlation was maximized between data and a reference artifact (the first artifact in the data). The second stage of FASTR [5] computed an average template for the cardio ballistic artifact. It subtracted this template from the center contaminated data by taking a moving window average of slice artifacts. QRS detection utilizes combined adaptive thresholding [11] and the Teager energy operator [27], followed by a correction algorithm to detect heartbeats robustly for false positives and negatives and remove the pulse artifacts by using an optimal basis set for the number of PCs 3.

Further, Harvard Automated Processing Pipeline (HAPPE) [14] is employed for noise-free time–frequency analyses using EEGLAB (MathWorks). MR and CB artifact corrected EEG data are subjected to the 0.1 Hz high-pass and 57 Hz low-pass filtering followed by ICA decomposition using all the EEG channels. The electrical noise is removed using the Clean Line program (Mullen, 2012) through the multi-taper regression approach implemented in EEGLAB. The bad channels are identified by evaluating the normed joint probability of the average log power of all the selected channels and removed for the probability of more than three standard deviations from the mean for further analyses. A wavelet-enhanced ICA (W-ICA) approach removes eye and muscle-generated artifacts, high-amplitude artifacts, and signal discontinuities from EEG data. This approach of W-ICA followed by ICA improves the resulting ICA decomposition of the EEG data [63]. A machine-learning algorithm MARA (Multiple Artifact Rejection Algorithm) [59] evaluates the ICA-derived components for automated component rejection for artifact probabilities greater than 0.5.

Further, artifact rejected data are segmented based on event markers. The artifact-corrected datasets were subsequently downsampled to 250 Hz and re-referenced to the common average reference and bandpass filtered from 1 to 48 Hz ((1–4) Hz for delta, (4–8) Hz for theta, (9–14) Hz for an alpha, (15–35) Hz for beta and (35–48) Hz for gamma)). Finally, all the participants' artifact corrected data are then segregated specific to the target, distractor, and fixation blocks. Further, simultaneously acquired fMRI data are preprocessed using Statistical Parametric Mapping version 12. The information is corrected for slice-timing differences, spatially realigned, and excluded if movement exceeds 3 mm. It follows by registering the functional scans to standard MNI template space. Further, artifact-corrected images are subjected to spatially smoothing with a 5*5*5 mm full-width half-maximum Gaussian kernel.

### Estimation of frequency-microstates and their optimization with task's cortical communications

**Fig. 2:**
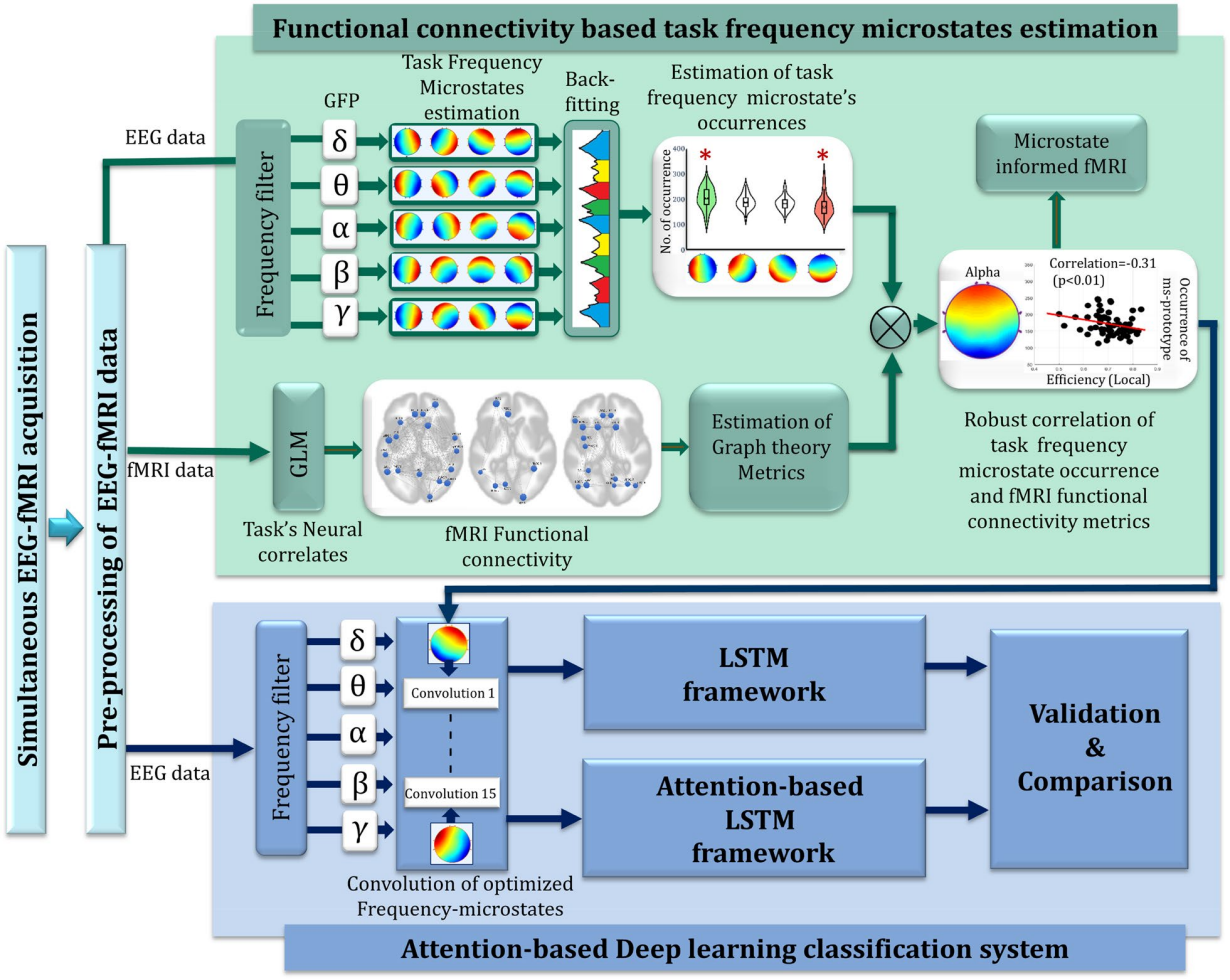
Methodological framework of the study

The present study develops a computational framework that classifies distinct task engagement's temporal global cortical communication through unique temporal EEG quasi-stable information that decodes the neural basis of the distant cortical communications. Figure 2 explains these processes in detail. The following section will elaborate on each one of these steps in detail.

At first, artifact-corrected EEG data are bandpass filtered to segregate it into frequency-band limited data comprising (1–4) Hz for delta, (4–8) Hz for theta, (9–14) Hz for an alpha, (15–35) Hz for beta and (35–48) Hz for gamma. Then, each frequency information's Global Field Power (GFP) is computed and subjected to the modified K-means clustering algorithm [46] to identify every frequency-microstate topographic prototype. A detailed description of this estimation is given in Additional file 1: Section S1. Then, each frequency-microstate prototype is back fitted in every individual's data and estimated re-expressed sequences of microstate classes. Finally, statistics about the sequence of microstate classes, such as their frequency of occurrence or average duration, are calculated. This quasi-stable frequency-microstate patterns elicitation information is subsequently mapped with global functional connectivity of each task engagement assessed from simultaneously acquired fMRI information.

Simultaneously, the task's neural sources are identified from simultaneously acquired fMRI information employing the General Linear Model-based (GLM) analysis. At the subject level analysis, BOLD responses of each task engagement (target, distractor, and fixation) are modeled by a canonical hemodynamic response function with temporal and dispersion derivatives with six realignment parameters for each run. In second-level GLM modeling, group average maps were computed using one-sample t-tests, cluster corrected (p < 0.05) across subjects. Subsequently, the second-level GLM model results for every task engagement are passed as regions of interest (ROI) to graph theory analysis to estimate the global/local functional connectivity [58]. A detailed description of this estimation is given in the Additional file 1: Section S2. Finally, the graph theory metrics such as global and local efficiency of the functionally connected regions are estimated for each subject, and the ROI-to-ROI connectivity matrix is thresholded at p-FDR < 0.05 in a two-sided analysis.

Finally, these tasks' global cortical communications information estimated from fMRI graph theoretical measures are utilized to optimize and identify the relevant cortical quasi-stable frequency EEG elicitations that correspond to the task engagement. For this purpose, the number of occurrences of each delta, theta, alpha, beta, and gamma EEG microstates of every individual during task engagements (target, distractor, and fixation) is subjected to the robust correlation with global functional connectivities metrics measured from the simultaneously measured fMRI information. This study further validated these significantly correlating frequency-microstates by subjecting them to the EEG-informed fMRI analysis [1, 19] and studying their neural mechanisms. Since the frequency microstates that correlate with fMRI functional connectivity metrics are different for target, distractor, and fixation, three separate EEG-informed fMRI models were constructed for every task engagement. A detailed description of this estimation is given in the Additional file 1: Section S3.

### Attention-based LSTM model and validation

**Fig. 3:**
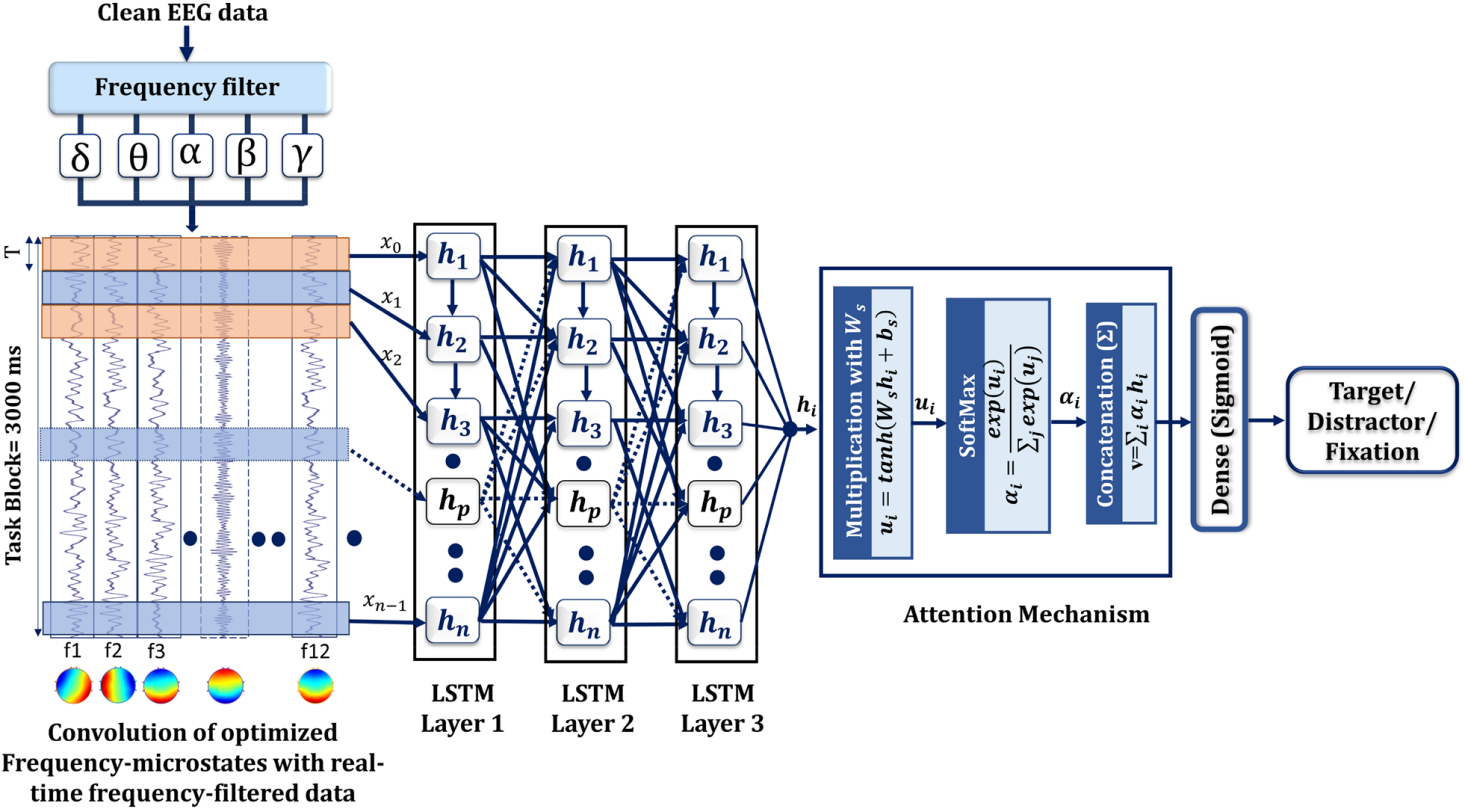
Schematic diagram of Attention-based LSTM deep learning framework adopted in the study. 'T' represents the temporal, sliding input sampling window size and is chosen between 180 to 750 ms/segment associated with the specific number of cells ranging from 4 to 16, based on the stability of transition probabilities of the optimized microstates

This study aims to develop an attention-based LSTM framework to classify the global cortical communication (estimated using fMRI functional connectivity optimized frequency quasi-stable oscillations) associated with global brain communications. For this purpose, each frequency-microstate strongly associated with fMRI functional connectivity measures was correlated with every individual preprocessed EEG information. Then, the feature vector consisting of the correlation value for each quasi-stable frequency microstates belonging to every task engagement is formed and used as a training, testing, and validation dataset for the attention-based LSTM deep learning model. The attention-based LSTM model employed in this study is illustrated in Fig. 3.

LSTM has been a popular recurrent neural network for learning sequential features in time-series data and classifying EEG information [41, 57]. However, the standard LSTM is incapable of detecting important parts for classification. The attention mechanism is recently conceptualized [55] and integrated with the LSTM framework to overcome this issue. The attention mechanism improves the ability of LSTM by concentrating on time fragments with the most discriminative information in EEG time-series. It helps to classify the data that involve remembering and aggregating feature embeddings in time-series information [65]. A detailed description of the LSTM model and attention mechanism is explained below.

The LSTM architecture consists of interconnected cells that hold the memory information through which the input data are processed to evaluate the output results. The cells have a common cell state, keeping long-term dependencies along the entire LSTM chain of cells. In LSTM, input information flow is controlled by the input gate (i_t_) and forget gate (${f}_{t}$) and allows the network to decide whether to forget the previous state (${C}_{t-1}$) or update the current state (${C}_{t}$) with new information. Further, each cell's result is modulated by an output gate (${o}_{t}$), which lets the cell compute its output based on the updated information. The function of LSTM cell architecture is calculated as:

1 $${\mathrm{i}}_{\mathrm{t}}=\upsigma \left({\mathrm{W}}_{\mathrm{i}} . \left[{\mathrm{h}}_{\mathrm{t}-1},{\mathrm{x}}_{\mathrm{t}}\right]+{\mathrm{b}}_{\mathrm{i}}\right),$$

2 $${\mathrm{f}}_{\mathrm{t}}=\upsigma \left({\mathrm{W}}_{\mathrm{f}} . \left[{\mathrm{h}}_{\mathrm{t}-1},{\mathrm{x}}_{\mathrm{t}}\right]+{\mathrm{b}}_{\mathrm{f}}\right),$$

3 $${\mathrm{C}}_{\mathrm{t}}={\mathrm{f}}_{\mathrm{t}}*{\mathrm{C}}_{\mathrm{t}-1}+{\mathrm{i}}_{\mathrm{t}}*\mathrm{tanh}\left({\mathrm{W}}_{\mathrm{c}} .\left[{\mathrm{h}}_{\mathrm{t}-1},{\mathrm{x}}_{\mathrm{t}}\right]+{\mathrm{b}}_{\mathrm{c}}\right), $$

4 $${\mathrm{o}}_{\mathrm{t}}=\upsigma \left({\mathrm{W}}_{\mathrm{o}} . \left[{\mathrm{h}}_{\mathrm{t}-1},{\mathrm{x}}_{\mathrm{t}}\right]+{\mathrm{b}}_{\mathrm{o}}\right),$$

5 $${\mathrm{h}}_{\mathrm{t}}={\mathrm{o}}_{\mathrm{t}}*\mathrm{tanh}\left({\mathrm{C}}_{\mathrm{t}}\right),$$

where $\sigma \left(x\right)=\frac{1}{1+{e}^{-x}}$ ,$\mathrm{tanh}\left(x\right)=\frac{2}{1+{e}^{-2x}}-1$. h_t_, C_t-1_ and x_t_ are the hidden state, cell state and input passed to the architecture at time step t. W_f_, W_i_, W_c_,W_o_ are the weights, and b_f_, b_i_, b_c_, and b_o_ are the biases.

#### Attention mechanism

The core concept of the attention mechanism is to simulate the human attention mechanism to improve the performance of deep learning [39] by utilizing the weighting parameters of the elements in the input sequence to generate the output sequence. It is carried out by multiplying the hidden output states by trainable weights, capturing more discriminative task-related features, and can be expressed as:

6 $${\mathrm{h}}_{\mathrm{i}}=\mathrm{LSTM}\left({\mathrm{s}}_{\mathrm{i}}\right), \mathrm{i}\in \left[1,\mathrm{ L}\right],$$

where h_i_ is the output hidden state vector for the ith LSTM cell corresponding to the ith input, and L is the number of cells in each recurrent layer of the LSTM network. To capture the importance of each hidden state, the attention mechanism is defined as follows:

7 $${u}_{i}=\mathrm{tanh}\left({\mathrm{W}}_{\mathrm{s}}{\mathrm{h}}_{\mathrm{i}}+{\mathrm{b}}_{\mathrm{s}}\right),$$

8 $${\alpha }_{i}=\frac{exp\left({u}_{i}\right)}{\sum_{j}exp\left({u}_{j}\right)},$$

9 $$\mathrm{v}=\sum_{i}{\alpha }_{i}{h}_{i},$$

where vector v is the attention layer's output, and Ws and bs are trainable parameters.

This study uses all 12 quasi-stable frequency microstates (correlated with task engagement's neural mechanisms) as input information to train attention-based LSTM models. As mentioned in the methods, 70 volunteers participated in the study and performed target identification, distractor identification, and fixation (rest). Each paradigm involved 32 blocks of the target, 62 blocks of the distractor, and 94 blocks of fixation. More distractors and fixation are needed to avoid the expectancy aspect of cognitive engagement. For this, 32-channel EEG is employed to acquire the data with a 5k sampling rate and subsampled to 250 Hz during preprocessing. Each task engagement block was carried out for 3 s; 750 feature vectors were staggered for every task block. The 32nd channel data are ECG, hence not included for processing. Accordingly, each volunteer will have a 750 × 31 dataset at every block. Equal numbers, 32 blocks from each target, distractor, and fixation are selected for training. Selecting distractor and fixation from many blocks is done randomly to avoid training bias. Hence, each volunteer had selected data with a size of 72,000 × 31 (24,000 × 31 for every task engagement). The preprocessed EEG data of the complete 70 volunteer set (50,40,000 × 31) are initially subjected to the correlation with all the 12 significant frequency microstates, resulting in feature vectors with the size of 50,40,000 × 12. Each feature vector consists of the correlation value for each one of the quasi-stable frequency microstate information belonging to every task engagement is formed. The feature vectors are combined across task blocks, and distinct segregation of input feature vectors belonging to every task block was explored. For this, the study independently employed different attention-based LSTM architectures in input layers with 4 to 16-LSTM cells. This enabled analyzing the effect of temporal, sliding sampling window width on the performance of deep learning architectures. As every task stimulus last for 3 s, choosing different deep learning architectures with LSTM nodes of 4 to 16 allowed the EEG sliding temporal window to be 750 ms to 180 ms. The final LSTM layer was ensued by an attention layer, which was succeeded by a fully connected layer with a sigmoid activation function to predict the probability of each task engagement. Finally, to estimate the efficacy of attention mechanisms in the original LSTM system, all attention-based LSTM architectures were compared with their attention counterpart. The tuning parameters were applied after each LSTM layer and optimized the weight matrix L2 regularization coefficient of each LSTM layer for optimization. Finally, hyper-parameter such as learning rate was tuned for the stochastic Adam optimizer. The final optimized tuned parameter that performed better is tabulated in Table 1.

**Table 1:** Final tuning parameter of LSTM model

Hyperparameters	Tuned parameters
Hidden layer size	256
Batch size	64
Training epoch numbers	1000
Rate dropout	Input Layer: 0, 1st LSTM Layer: 0.2, 2nd LSTM Layer: 0.1, 3rd LSTM layer: 0.2
Recurrent depth	3
Learning rate	0.001

#### Validation

Finally, the attention-based LSTM architecture is validated by employing a tenfold cross-validation approach with no overlap of training and testing segments. True positive (TP), true negative (TN), false negative (FN), and false positive (FP) were used to calculate the performance metrics. They are formulated as Precision = TP/(TP + FP), Accuracy = (TP + TN)/(TP + TN + FP + FN) and Recall = TP/(TP + FN).

## Results

The present study presents the time-series computational frameworks that classify different task engagement based on temporal modulation of distant brain communications through optimized frequency EEG microstates. The frequency EEG microstates were optimized by associating them with simultaneously eliciting distant hemodynamic functional connectivity measures. Further, the study explored the EEG-informed fMRI approach employed to understand the insights into the neuronal mechanisms related to frequency microstates that correlate with global task communications. The following sections present the results of each of the above steps in detail.

### Frontal–parietal–temporal regions interaction during task engagements

The neural sources associated with each task engagement are assessed through fMRI GLM models with the double-sided *t*-test of *p* < 0.5, FDR corrected. The results suggest that target engagement enhanced the hemodynamic response in the frontal [frontal orbital cortex (FOC), frontal pole (FP), superior frontal gyrus (SFG)], parietal [angular gyrus (AG), Precuneus cortex (PC)], and temporal [Inferior and middle temporal Gyrus (ITG, MTG)] cortices. Further, it involves significant engagement of the cingulate gyrus (CG), lateral occipital cortex, occipital pole (OP), paracingulate gyrus, and insular cortex (IC) regions. The neural correlates of distractor and fixation have also revealed significantly different intercortical engagement. The detailed list of neural correlates of each task engagement is tabulated in Additional file 1: Table S1.

This distinct inter-cortical communication during each task engagement is further supported by the graph theoretical functional connectivity metrics such as global (GE) and local efficiency (LE). The target engagement significantly engaged inter and intra-cortical communication at frontal cortex (FP [GE:0.893, LE:0.89], FOC [GE: 0.886, LE:0.89], and SFG [GE:0.886, LE:0.894]), parietal cortex (left AG [GE:0.872, LE:0.893], right AG [GE:0.9, LE:0.887], PC [GE:0.87, LE:0.894]) and temporal cortical regions (ITG [GE: 0.88, LE: 0.889], MTG [GE:0.889, LE: 0.891], temporal occipital fusiform cortex (TOFC) [GE:0.88, LE:0.89]) at *p* < 0.05 with FDR correction. Functional connectivity elicitation remained distinct for the target detection task and had minimal overlap with other task engagement. Notably, frontoparietal and frontotemporal interaction engagement were distinct for target, distractor, and fixation engagement. The detailed information on graph-theoretical measures estimated for each task engagement is given in Additional file 1: Section S2.

### Hemodynamic functional connectivity optimization of quasi-stable frequency-microstates

**Fig. 4:**
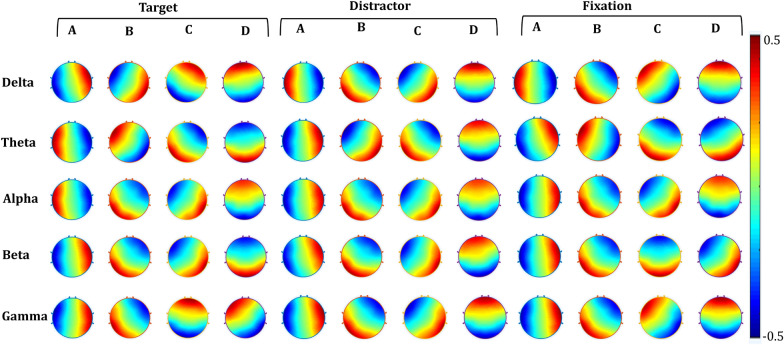
A topographical representation of frequency-microstates for the target, distractor, and fixation

Frequency microstates estimation revealed four dominant microstates for every EEG frequency band of each task engagement. Figure 4 illustrates spatial topographical patterns of frequency-microstate topography associated with every task engagement.

**Fig. 5:**
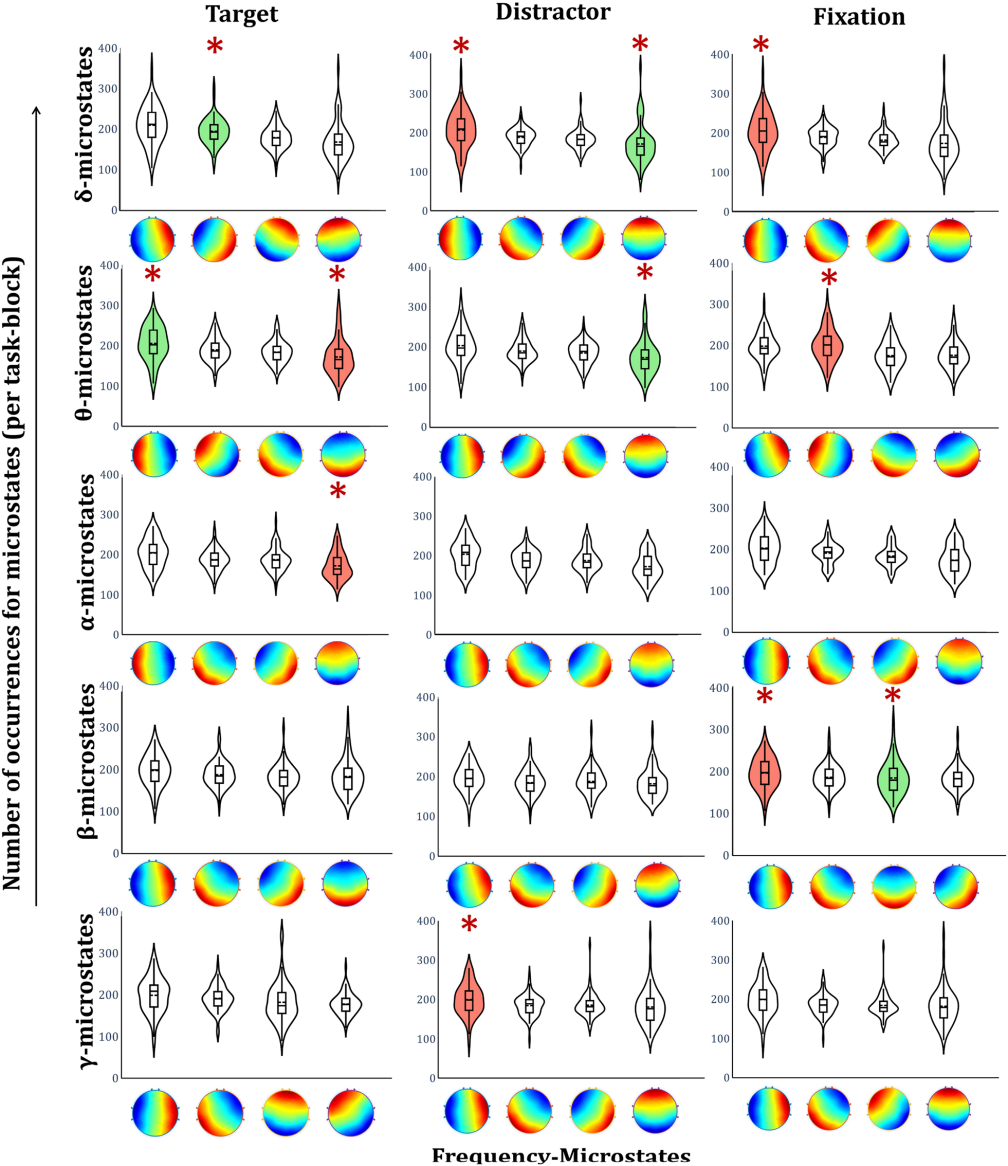
Estimated metrics of frequency-microstates. The violin graph plots the mean occurrence of each frequency-microstates for each frequency band. The violin plot's green and red color shades reveal the correlation (positive and negative) of the number of occurrences of quasi-stable elicitation with the task's fMRI functional connectivity measures. The red star on violin plots specifies the significance (*p* < 0.01)

**Fig. 6:**
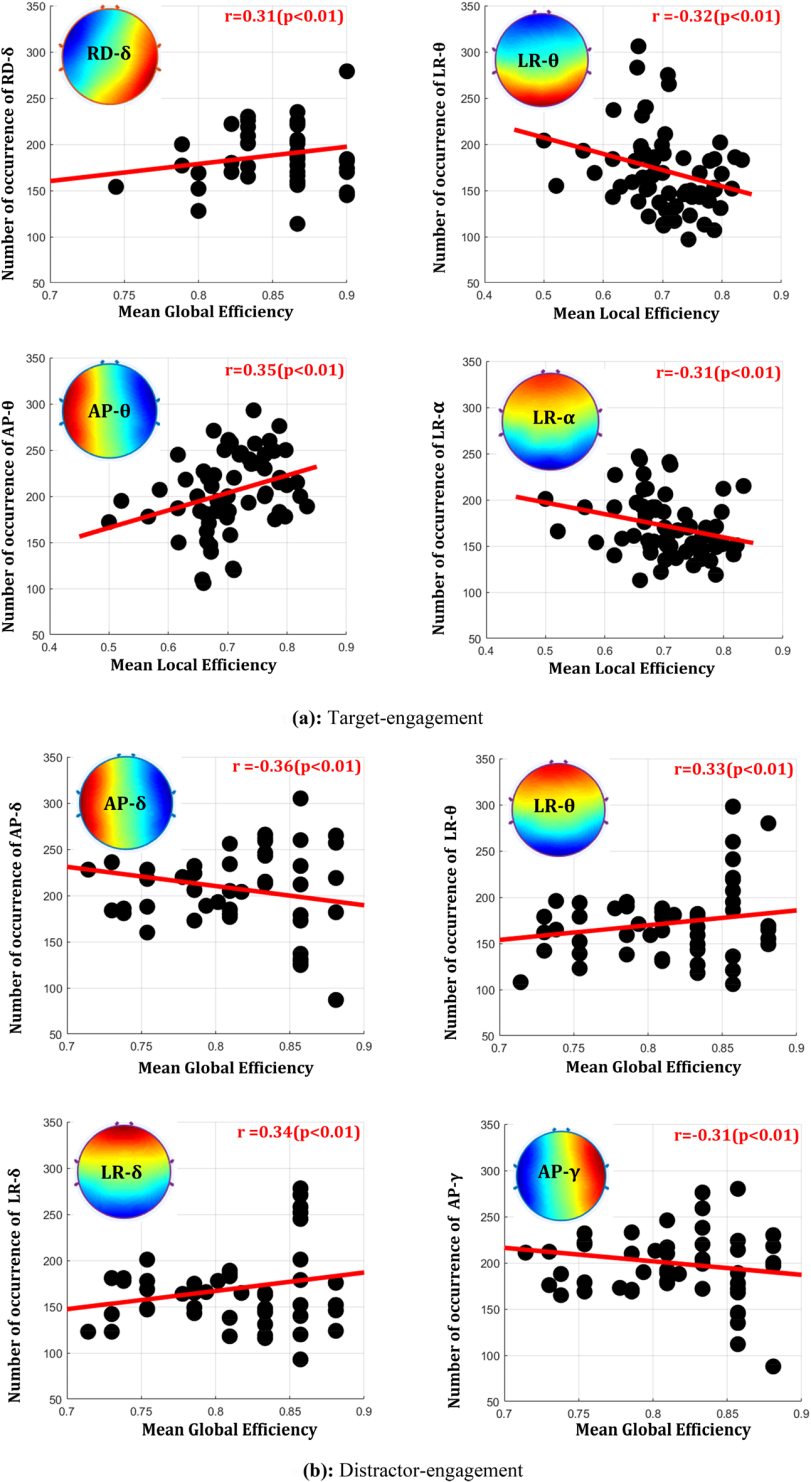

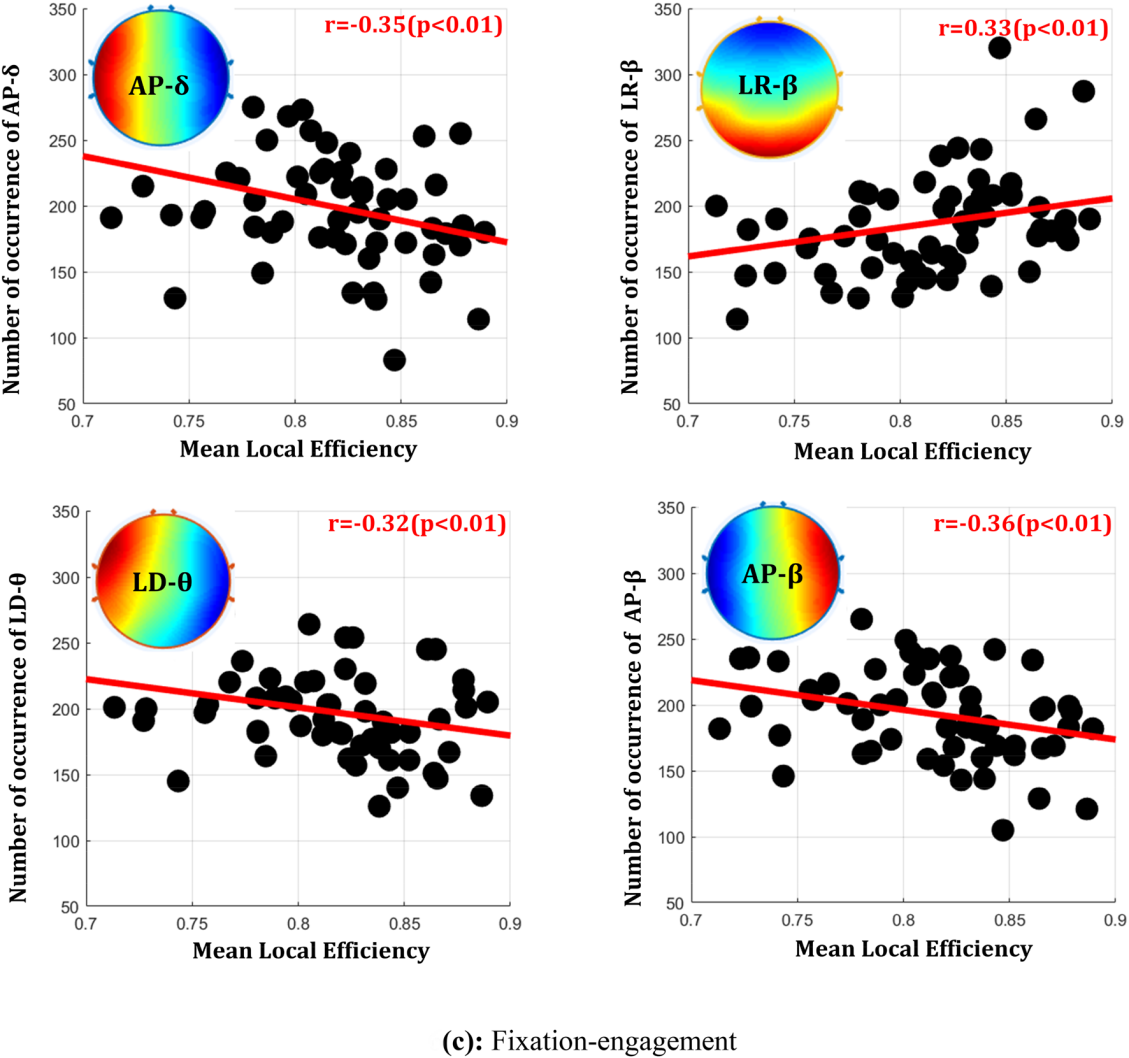
Significantly associated, optimized, frequency-microstates with graph-theoretical measures of **a** target, **b** distractor, and **c** fixation task engagement. Frequency microstates are labeled as anterior–posterior (AP), left–right (LR), left diagonal (LD), and right diagonal (RD)

Further, Fig. 5 presents the mean number of occurrences of all frequency microstates and highlights 12 specific frequency microstates whose number of occurrences is robustly correlated (+ ve correlation: green, −ve correlation: red) with the fMRI functional connectivity measures. The detailed correlation information of frequency microstates with graph-theoretical measures of every task engagement is illustrated in Fig. 6. In this study, based on the microstate's green color band direction, they are labeled as anterior–posterior (AP), left–right (LR), left diagonal (LD), and right diagonal (RD) microstate prototypes. For example, the delta microstate with a green band traveling between anterior–posterior is called the "anterior–posterior delta microstate." During target engagement, the number of occurrences of right diagonal delta-microstate positively correlates with the global efficiency of fMRI functional connectivity measures. On the other hand, the local efficiency of fMRI connectivity measures correlates negatively with both left–right theta and alpha microstate occurrences and positively with anterior–posterior theta microstate.

### Neurovascular analysis of optimized frequency microstates: EEG-informed fMRI analysis

Engagement of task generally elicits local neural clustering (high-frequency quasi-stable oscillations) at distinct brain regions, responsible for efficient local information processing, together with distant cortical intercommunication to facilitate global communication (low-frequency quasi-stable oscillations). The neuro-vascular analysis through EEG-informed fMRI explains these insights through synchronizing neural information (optimized quasi-stable EEG oscillations) with hemodynamic information (vascular) elicited from a specific task engagement. For this purpose, each of the 12 optimized frequency microstates is processed in independent EEG-informed fMRI models, which modeled each microstate as separate regressors (*p* < 0.01, FDR corrected) to estimate their neuro-vascular information. Additional file 1: Fig. S1a–c shows neuro-vascular information of each 12 optimized microstates.

**Fig. 7:**
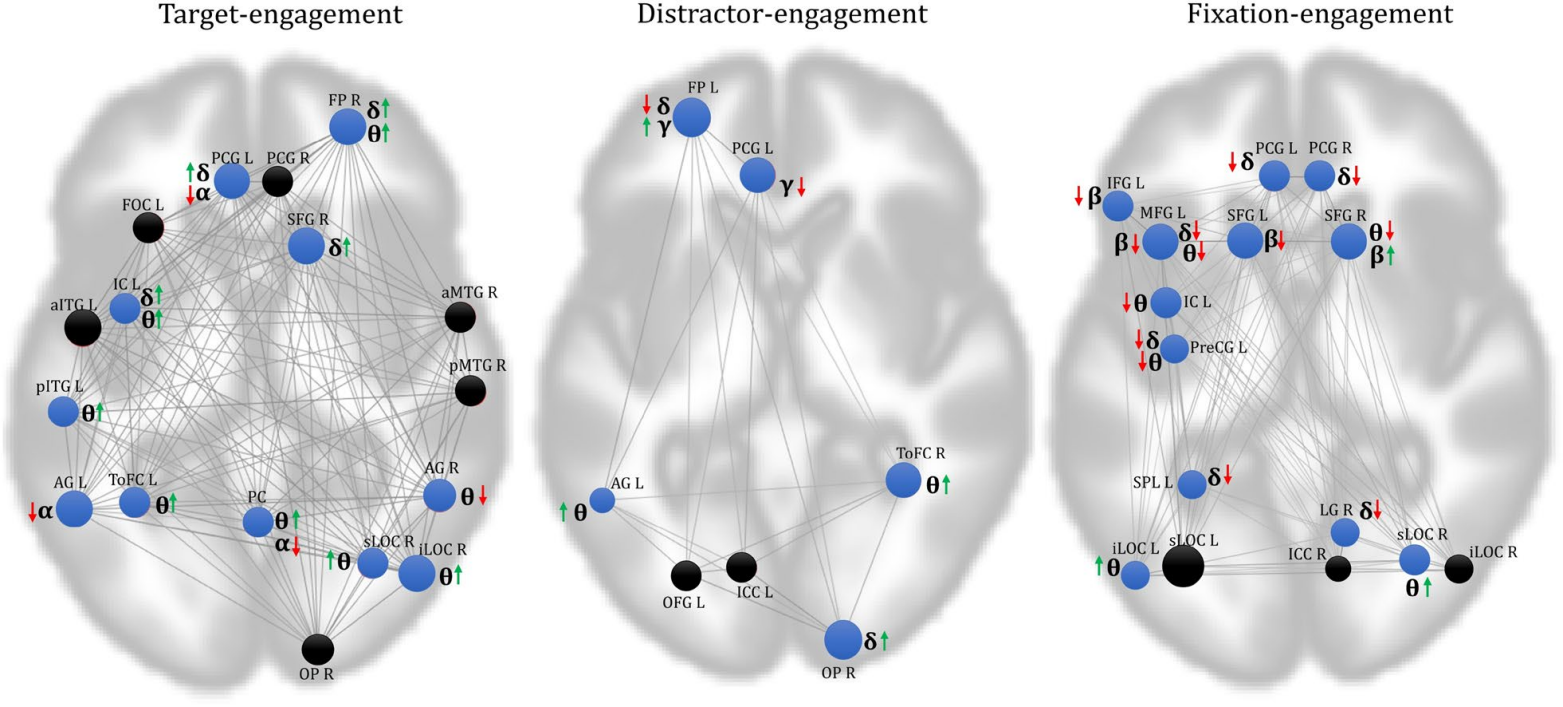
Neuro-vascular coupling of each significant, fMRI functional connectivity optimized frequency-microstates of every task. The Regions shown are neural correlates observed for every task. The neuro-vascular association of optimized frequency microstate with each region is drawn through the arrow next to it (Synchronization: Up green arrow, De-synchronization: Down Red arrow). The area colored black has no neurovascular association with any of the optimized frequency microstates

Figure 7 shows the neuro-vascular coupling of each significant frequency-microstate at the neural correlates of the respective task engagement and the associated functional connectivity. Target engagement elucidated the right-diagonal delta-microstate synchronously with the BOLD response in the frontal cortex. Further, anterior–posterior and left–right theta-microstates are found to synchronize with the BOLD response of frontal, temporal, parietal, and occipital regions except for right-lateralized SFG and AG. The role of theta-microstate in BOLD-synchronization of these regions characterizes its significant association with target engagement. The alpha-microstate is observed to de-synchronize with the parietal, PCG's BOLD response, and synchronize with the frontal, occipital region during target engagement. Our findings also reveal the effects of multiple frequencies on specific brain regions, such that the BOLD response of FP, PCG, SFG, IC, PC, and AG are modulated with delta, theta, and alpha-microstates. Specifically, the study observes the de-synchronization of alpha-microstate with the delta and theta microstates such as PCG, SFG, PC, and synchronization between delta and theta microstates as IC and FP. Hence, the relationships mentioned above reveal that the multi-frequency interactions modulate the BOLD response of task-engaged brain regions.

### Performance of deep attentional LSTM model

**Fig. 8:**
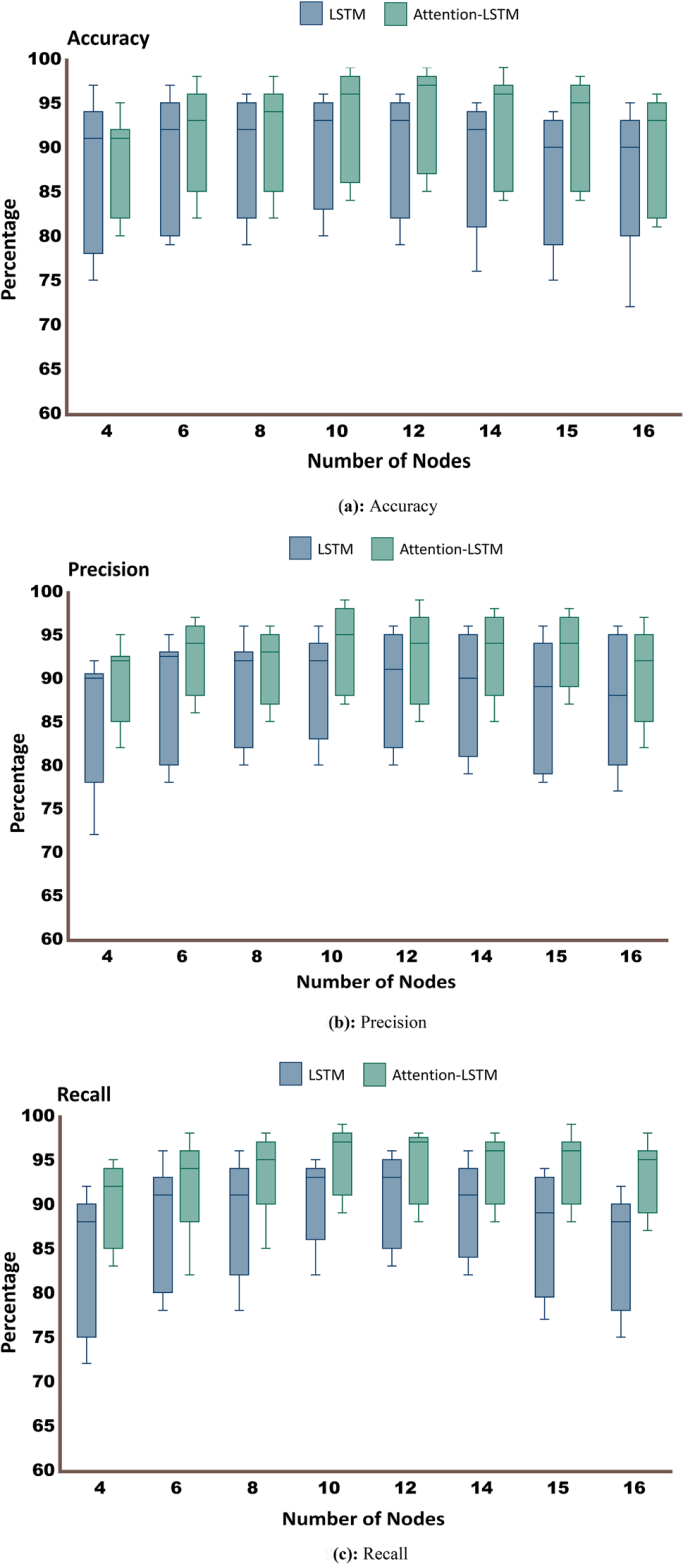
Comparison of performance of the deep learning frameworks. **A** Accuracy, **B** precision, **C** recall. Each architecture was independently optimized with 4, 6, 8, 10, 12, 14, 15 and 16 LSTM input cells for temporal EEG sampling of 750, 500, 375, 300, 250, 214, 200 to 180 ms/segment, respectively

Figure 8 compares the performance metrics, precision, accuracy, and recall of both deep learning models (LSTM and attention-LSTM). As observed, LSTM combined with attention performed significantly better in all three-performance metrics than the traditional LSTM model. The benefit of choosing different temporal sampling window sizes (180 ms/segment to 750 ms/segment) of input feature vectors by choosing 16 to 4 LSTM cells in input layers were analyzed within LSTM and attention-LSTM architectures. The results reflected the significant improvement in performance while using ten nodes (300 ms /segment), and a substantial decline in performance metrics is observed for 16 (180 ms/segment) nodes.

## Discussion

The present study brings more insights into understanding and optimizing frequency microstate information estimated from cortical, coarse EEG information with distant fMRI functional connectivity measures associated with different task engagements. It utilizes them to train a time-series of deep learning frameworks. The study employed an attention-based stacked LSTM for effectively remembering and aggregating feature embeddings in the time-series classification of stacked temporal dynamics of the frequency microstate quasi-stable patterns. The results reveal that hemodynamic functional connectivity optimized quasi-stable frequency microstates enable attention-based LSTM algorithm to better classify target engagement, distractors, and fixation. Further, the study employed EEG-informed fMRI to understand the optimized frequency microstates' neuro-vascular insights and their association mechanisms. The following sections discuss these observations in detail.

### Association of distinct frequency microstates with each task's cortical functional connectivity

The study observed four distinct cortical frequency-microstates elicitations for each of three different task engagements (thus, 12 distinct frequency microstates) independently correlated strongly with their hemodynamic functional connectivity measures. Non-overlap of association of these frequency microstates clearly states the underlying difference in distant cortical communications associated with each task engagement. In addition, the study also has observed a distinct neuro-vascular functional association of slow and faster oscillations in the brain regions involved in each task's cortical communications. Some essential observations of these multi-frequency quasi-stable EEG frequency associations with each task's distant neural interactions are summarized below.

### Association of quasi-stable microstates oscillations with global/local neural engagements

Complex cognitive engagement requires global interactions of different brain regions enabling the large-scale integration of local neural information. The integration of neural engagement of spatially distant areas constituting the large-scale networks is primarily molded with low-frequency synchronized oscillations due to their long-range communications and integrative roles in various brain functions.

Further, the strength of large-scale networks has also been found to be highest for lower frequencies and seen gradually decreasing with increases in the frequency range [15, 32, 60]. Therefore, the dominance of large-scale networks for the lower range of frequencies confirms their functional significance. The present study's results (Fig. 6) distinctly revealed synchronization (positive correlation) of the slow frequency oscillations with global, distant brain communication and de-synchronization (negative correlation) with local neural elicitation across all the task engagement. Similarly, the high-frequency quasi-stable oscillation distinctly synchronized with the elicitation of local neural engagement and desynchronized with global communications across all the task engagements. A similar observation (Fig. 7) is revealed in the neurovascular coupling from EEG-informed fMRI analysis at the brain regions engaged in all tasks. They demonstrated significant de-synchronization between slow and faster quasi-stable oscillations in most brain regions involved during every task engagement. Further, the region-wise neuro-vascular insights brought slower quasi-stable oscillations associations with large-scale frontoparietal and frontotemporal functional networks. These observations are supported by the proposal of Polich et al. [47] and Harper et al. [17], explaining the role of delta and theta band activity underlying the frontoparietal and frontotemporal functional networks.

The accumulating literature suggests the association of local neuronal processing with the global cortical communication between neural assemblies by coupling multiple oscillatory frequencies [10] and referred to as cross-frequency coupling. The most well-studied example of cross-frequency coupling is the theta-gamma coupling, which explains the engagement of gamma frequency in certain phases of theta cycles. Further, [50] suggested that low-frequency oscillations may be essential in engaging gamma rhythms during attention. [33] discussed the relationship of alpha and theta frequency oscillations in the cortex and revealed the possibility of the theta-gamma code's contribution to memory and sensory processes. Thus, the functional interaction of more extensive networks oscillating at lower frequencies and local neuronal ensembles oscillating at higher frequencies has been revealed for cortical communication and integration [13, 29, 33]. Hence, the studies mentioned above better understand the modulation of faster oscillations from the slow EEG oscillations during cognitive engagements.

The present study's quasi-stable cortical oscillation's neuro-vascular insights and their association with local and global neural information are consistent with these studies and explain the role of multi-frequency interactions in explaining the complex cognitive engagements' neuronal mechanisms.

### Performance of attention-based deep learning system in classifying time-series quasi-stable cortical frequency EEG information

The proposed deep learning architectures (Fig. 3) revealed comparable accuracy, precision, and recall rate for classifying target detection task engagements in Fig. 8. However, two primary aspects distinguished the performance of each proposed deep learning architecture. They are the temporal sampling window and incorporation of the attention mechanism.

#### Temporal sampling window of quasi-stable frequency information optimizes the performance of deep learning architectures

Temporal sampling window size plays a significant role in achieving the best classification accuracy of deep learning architectures. This crucial aspect of time-locked microstates events facilitated a better temporal data handling of multi-frequency interaction in the cognitive task's neural engagements. The present study employed multiple windows of temporal sampling (four nodes: 750 ms, six nodes: 500 ms, eight nodes: 375 ms, 10 nodes: 300 ms, 12 nodes: 250 ms, 14 nodes: 214 ms, 15 nodes: 200 ms, and 16 nodes: 180 ms) to explore optimized, quasi-stable microstate's ability to classify task engagement. The highest classification accuracy, up to 99% for 10- and 12-node attention-based architecture, confirms the temporal dynamics of quasi-stable frequency oscillations optimally with 300 ms and 250 ms. Several target identification-related EEG studies support this observation [6–8, 43, 51] revealed that the peak of task event-related potentials following the stimulus onset in between 100 and 300 ms at multiple. Specifically, a recent EEG study [17] showed event-related synchronization of theta and delta bands occurring around 300 ms after the onset of target stimuli.

#### Effect of attention mechanism with traditional LSTM architectures

The overall better performance of all the deep learning architectures justified the optimization of quasi-stable frequency-microstate information. However, the attention mechanisms further improved this in the deep learning system. The combined effect of attention phenomena and the LSTM architecture allowed the deep learning architecture to dynamically emphasize the task-relevant neural information in the time-series sequence of EEG data and give less attention to other irrelevant information. The improvement in the performance of the attention-based LSTM system can be seen precisely in Fig. 8. The plots suggest that the attention mechanism is optimum for extracting the most relevant task neural features and improves the LSTM's performance compared to independent LSTM.

## Significance of the study

There is extensive literature (Additional file 1: Table S2) on employing a deep learning approach to decode task engagement using EEG elicitations. However, most of those works are restricted to the sensory–motor tasks (hand, leg movements, imagery tasks) whose engagement can be localized in a few cortical regions. However, minimal research is engaged to decode the cognitive task engagement's functional connectivity of distant and distinct cortical engagement using the deep learning framework. Further, despite many research studies that microstates are a promising neural signature, their association with the neural mechanisms of task engagement is still not clearly understood [26, 53, 54]. In addition, not many works in the literature explain the quasi-stable nature of the different EEG frequency oscillations either. The present work employs attention-based LSTM architecture to decode the temporal dynamics of cognitive task engagement through fMRI functional connectivity optimized frequency microstates. Recently, [52] investigated the temporal dynamics of traditional microstates using recurrent neural networks. However, their work did not address the quasi-stable frequency microstate's neural mechanism and modulation during task engagement. Our present work further brings more insights into the attention mechanism's ability to improvise the classification of cognitive task engagement based on the optimized neural signatures. To our knowledge, the present study is one of the few works that employs simultaneous EEG–fMRI information to optimize the neural signatures for improvising the performance of deep learning architectures.

## Conclusions

The present study proposes an attention-based deep learning framework that processes temporal dynamics of the 12 distinct, fMRI functional connectivity optimized, quasi-stable frequency microstates to classify different cognitive task engagement. It further utilizes neurovascular insights of these optimized frequency microstates through EEG-informed fMRI analysis to understand the local and distant cortical interaction revealed by the optimized frequency microstate. This optimized neural information was passed as input at distinct temporal samplings windows to train and validate the attention-based LSTM architecture. The results suggest that the classification accuracies of the attention-based LSTM architectures were better than the traditional LSTM architectures due to the ability of the attention mechanisms in deep learning systems to localize temporal feature information. Notably, the attention-based LSTM model with 250 ms per segment and 300 ms per segment temporal sampling revealed a higher classification accuracy than other architectures. Hence, the study demonstrates an attention-based deep learning framework to perform a robust classification of complex, distant cortical engagement and communication caused by cognitive task engagements based on the novel, quasi-stable frequency microstates.

## Supplementary Information

**Additional file 1. **Estimation of frequency-microstates. Estimation of global functional connectivity from simultaneously acquired fmri information. Optimizing frequency-microstate elicitations with fmri functional connectivity measures and their validation through microstate informed fmri. Additional figures and tables.

## Data Availability

The datasets used and/or analyzed during the current study are available from the corresponding author on reasonable request.
