# Supplementary material for: Hemodynamic functional connectivity optimization of frequency EEG microstates enables attention LSTM framework to classify distinct temporal cortical communications of different cognitive tasks
Source: Brain Inform. 2022 Oct 11;9(1):25. doi: 10.1186/s40708-022-00173-5 (PMC9554110; doi:10.1186/s40708-022-00173-5)
Supplement: Supplementary file 1 — Additional file 1. Estimation of frequency-microstates. Estimation of global functional connectivity from simultaneously acquired fmri information. Optimizing frequency-microstate elicitations with fmri functional connectivity measures and their validation through microstate informed fmri. Additional figures and tables. [file 40708_2022_173_MOESM1_ESM.docx]

**Additional file 1**

**S.1 Estimation of frequency-microstates**

For the estimation of EEG frequency-microstates, at first, artifact corrected EEG data ($X_{Preprocessed}$) is band-pass filtered to segregate it into frequency-band limited data comprising of delta (δ), theta (θ), alpha (α), beta (β), and gamma (γ) ranges.

$X_{filtered\_band}=FIR\left[ X_{Preprocessed} \right]$ .(1)

$FIR\left[ X_{Preprocessed} \right]=\left\{ \begin{matrix} 0, for {0\leq f\leq f}_{c1} \\ X_{Preprocessed,} {{for f}_{c1}\leq f\leq f}_{c2} \\ {0, for f}_{c2}\leq f\leq\pi\end{matrix} \right\}$ .(2)

Where$f_{c1}$ and $f_{c2}$are cut-off frequencies defined for each frequency band. The band-specific EEG data can be expressed as follows:

$X_{\delta}= X_{filtered\_band}\left\{ {f_{c1}=1,f}_{c2}=4 \right\}$ .(3)

$X_{\theta}= X_{filtered\_band}\left\{ {f_{c1}=4,f}_{c2}=8 \right\}$ .(4)

$X_{\alpha}= X_{filtered\_band}\left\{ {f_{c1}=9,f}_{c2}=14 \right\}$ .(5)

$X_{\beta}= X_{filtered\_band}\left\{ {f_{c1}=15,f}_{c2}=35 \right\}$ .(6)

$X_{\gamma}= X_{filtered\_band}\left\{ {f_{c1}=36,f}_{c2}=48 \right\}$ .(7)

Global Field Power (GFP) is computed for each frequency band-limited EEG data for each time point. GFP is equivalent to the standard deviation of the electric voltage for a given time frame used to identify changes in the overall level of activation of the brain. It is expressed as:

${GFP}_{filtered\_band}=\frac{\sqrt{\sum_{i=1}^{M} \left( X_{i,filtered\_band}-\bar{X_{i,filtered\_band}} \right)^{2}}}{M}$ .(8)

Where M is the number of channels, $X_{i,filtered\_band}$ is the voltage at electrode i for selected frequency band and $\bar{X_{i,filtered\_band}}$ is the average voltage of electrode of map.

GFP peaks are extracted for each frequency band with a minimum peak distance of 10 ms using the above equations. The minimum distance between GFP peaks ensures the distinctness of peaks. Aggregated GFP peak data for each band from 70 volunteers are subjected to the predefined K-means clustering algorithm. A modified K-means clustering algorithm (Pascual-Marqui et al., 1995) is used to identify the microstate topographic clusters from segmented data since it ignores the polarity of the EEG topography. The microstate classes are observed as ‘directions’ in a multi-dimensional topographical space, and these microstate activations quantify the distance of EEG signal along a microstate-orientation at a given time point.

$x_{n}={Az}_{n}+\in_{n}$ for n=1 : N .(9)

Where $x_{n}$ is the n^th^ time sample of EEG, A is prototypical maps for K clusters, $z_{n}$ is microstate activations for the n’^th^ time sample and $\in_{n}$ is the error term. The microstate label of an EEG sample is found as the microstate index, k, that minimizes the orthogonal squared Euclidean distance,

$l_{n}=arg \min_{k} \left\{ d_{kn}^{2} \right\}$, .(10)

$d_{kn}^{2}=x_{n}^{T} .x_{n}-\left( x_{n}^{T}.a_{k} \right)^{2}$ .(11)

In this way, the algorithm groups it into a small set of classes based on topographic similarity and estimates for each microstate class a topographical prototype. Clustering generated four microstates maps for each frequency band (delta, theta, alpha, beta, and gamma). After obtaining the microstate prototypes for each frequency band, the topography of each EEG sample is assigned to one of the microstate prototypes in back-fitting, it is most similar with, and the EEG signal for each frequency band is re-expressed as a sequence of microstate classes.

After estimating the microstate prototypes, measures of fit (Murray et al., 2008) are calculated to make a qualitative decision to fit EEG data to the microstate prototypes based on the Global explained variance (GEV) and cross-validation criterion (CV). Global explained variance (GEV) measures the similarity of each EEG sample with the assigned microstate prototype. GEV of all samples are aggregated as a measure of fit for a microstate segmentation and calculated as:

$GEV=\frac{\left( Corr\left( x_{n},a_{ln} \right).{GFP}_{n} \right)^{2}}{\sum_{n^{'}}^{N} {GFP}_{n^{'}}^{2}}$ .(12)

Subsequently, the cross-validation criterion (CV) measures the residual noise and is calculated as:

$CV=\hat{\sigma}^{2}. \left( \frac{M-1}{M-K-1} \right)^{2}$ .(13)

$\hat{\sigma}^{2}=\frac{\sum_{n}^{N} x_{n}^{T} x_{n}-\left( a_{ln}^{T}.x_{n} \right)^{2}}{N\left( M-1 \right)}$ .(14)

Where M is the number of channels, N is the number of time samples and $\hat{\sigma}^{2}$ is an estimator of the variance of the residual noise.

The assignment of microstate prototypes to EEG samples are measured using a global map

dissimilarity (GMD) index, which is a distance measure that is invariant to the strength of the signal and instead only looks at how similar the topographical maps look and calculated as:

$GMD=\frac{\left\| \frac{x_{n}}{{GFP}_{n}}-\frac{x_{n^{'}}}{{GFP}_{n^{'}}} \right\|}{\sqrt{M}}$ .(15)

Finally, statistics about the sequence of microstate classes, such as their frequency of occurrence or average duration, can be calculated. As the study primarily aims to identify the frequency-microstate that corresponds to the distant cortical communication caused by the task engagement, the number of occurrences of each frequency-microstates during each task engagement (target, distractor, and fixation) has been estimated. The number of times a frequency-microstate elucidated in one second in every individual’s back fitted frequency EEG information is estimated as the number of occurrences. This quasi-stable frequency-microstate patterns elicitation information is subsequently mapped with neural mechanisms and functional connectivity of each task engagement assessed from simultaneously acquired fMRI information.

**S.2 Estimation of global functional connectivity from simultaneously acquired fMRI information**

The neural correlates are identified from the second level of the GLM model to estimate distinct and distant cortical communication among task engaged brain regions, and subsequently subjected as regions pf interest (ROI) to the graph theory-based functional connectivity analysis. The CONN toolbox (Whitfield-gabrieli & Nieto-castanon, 2012) has been employed for this purpose. At first, the noise in fMRI information was reduced through the anatomical CompCor approach (Behzadi et al., 2007), which extracts principal components (PCA) from white matter and cerebrospinal fluid BOLD time series. These components were further added as confounds in the denoising step of the CONN toolbox. Each of the defined temporal confounding factors is subsequently regressed from the BOLD time series at each voxel, and the resulting residual time series are band-pass filtered. Linear detrending was additionally performed. Subsequently, the second-level GLM model results for every task engagement are passed as Regions of Interest (ROI) to graph theory analysis. The graph theory metrics such as global and local efficiency of the functionally connected regions characterizing the structural properties of the estimated ROI-to-ROI functional connectivity networks are estimated. Each subject-specific ROI-to-ROI connectivity matrix is thresholded at p-FDR<0.05 in a two-sided analysis and characterizes a graph with nodes associated with ROIs and edges associated with the strength of functional connectivity among these ROIs. For each node in a graph, global efficiency is measured as the average inverse shortest path distance from each node to all other nodes in the graph, and local efficiency is measured as the average global efficiency across all nodes in the local subgraph of each node. Both graph theory measures (global and local efficiency) are estimated at p-FDR<0.05 in a two-sided analysis.

**S.3 Optimizing frequency-microstate elicitations with fMRI functional connectivity measures and their validation through microstate informed fMRI**

The brain-computer interface technologies are primarily based on surface EEG information and fundamentally aim to decipher neural engagement and distant cortical communications caused by task engagement. However, getting these information pieces from the surface EEG is a more complex research question due to the volume conduction and superposition of neural activation as they travel through the intracranial regions. This study explored whether cortical quasi-stable frequency elicitation could be used as a task engagement neural marker. For this purpose, the number of occurrences of each delta, theta, alpha, beta, and gamma EEG-microstates of every individual during task engagement (target detection, distractor detection, and fixation identification) is subjected to the robust correlation analysis (Pernet et al., 2013) with functional connectivity metrics (global and local efficiencies) measured from the simultaneously measured fMRI information. The significantly correlating frequency microstates with the fMRI functional connectivity metrics are neural signatures associated with distinct and distant cortical communication caused by the associated task engagement.

This study further validated these significantly correlating frequency microstates by subjecting them to the EEG-informed fMRI analysis (Abreu et al., 2018; Huster et al., 2012) and studied their neural mechanisms. Since the frequency microstates that correlates with fMRI functional connectivity metrics are different for target, distractor, and fixation, three separate EEG-informed fMRI models were constructed for every task engagement. For this purpose, at first, each significantly correlating frequency-microstates is subjected to the back-fitting correlation with every volunteer's preprocessed, filtered EEG frequency information. Then, this correlation information is constructed for every 3 seconds to match simultaneously acquired fMRI information and passed as separate regressors in the EEG-informed fMRI model.  The first level analysis is performed using SPM 12. The EEG regressors are introduced as parametric modulators of the task execution period in the GLM design matrix, convolved with canonical HRF, the temporal-dispersion derivatives, and six realignment parameters for each run. At the first level, t-contrasts are calculated for each parametric modulator for frequency microstate. Subsequently, these first-level contrast images are subjected to group-level random-effects analysis using a robust regression toolbox (Wager et al., 2005). The robust regression toolbox uses robust iteratively reweighted least squares (IRLS) to identify and down-weight potential outliers that can cause an increase in false positives and false negatives. Effects observed at P<0.05 and surviving a false discovery rate (FDR) correction at the cluster level at P(FDR)<0.05 are reported as significant for fMRI analyses.

**Additional file 1 Figures:**


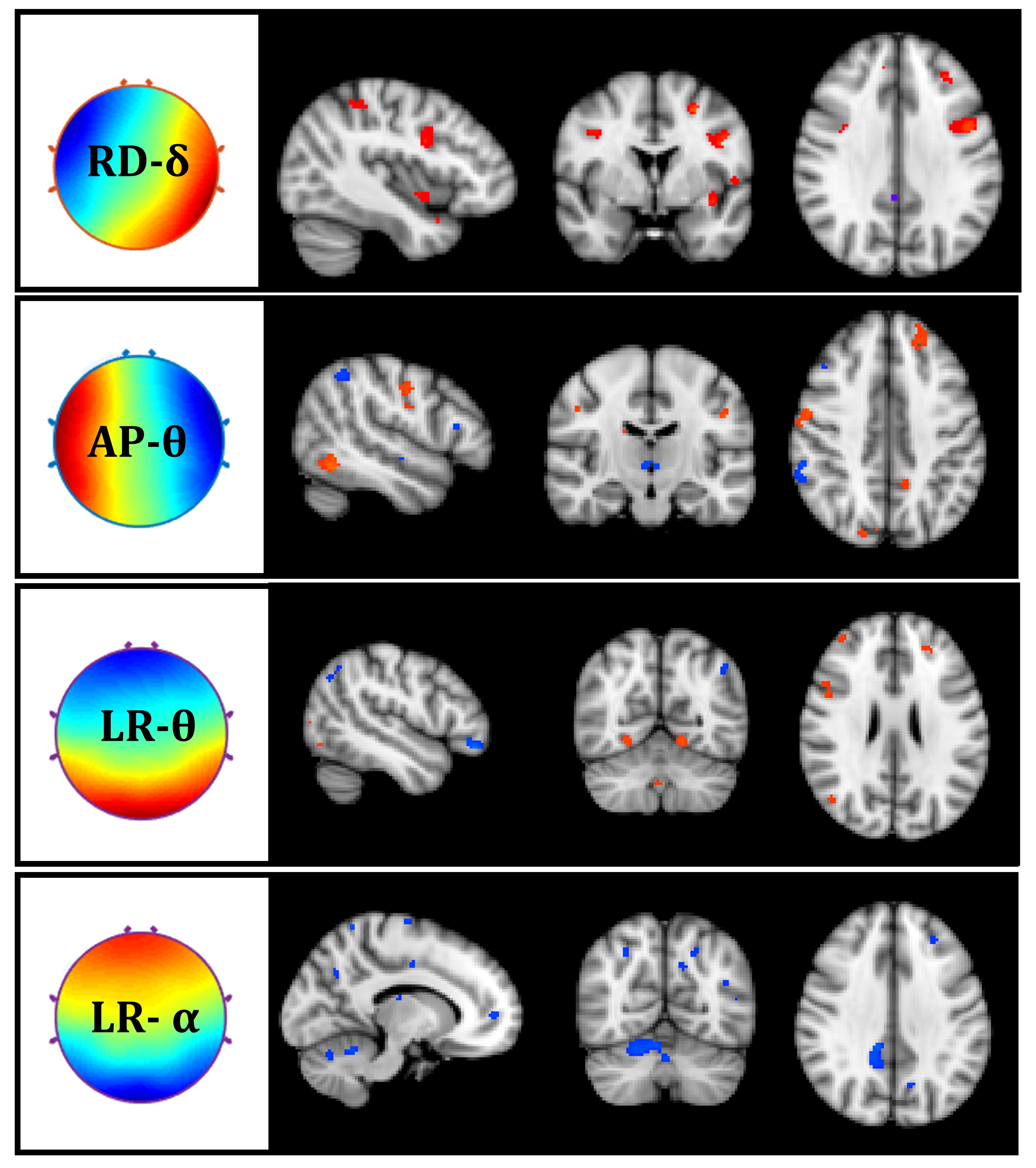


**Figure S1(a): Target-engagement**


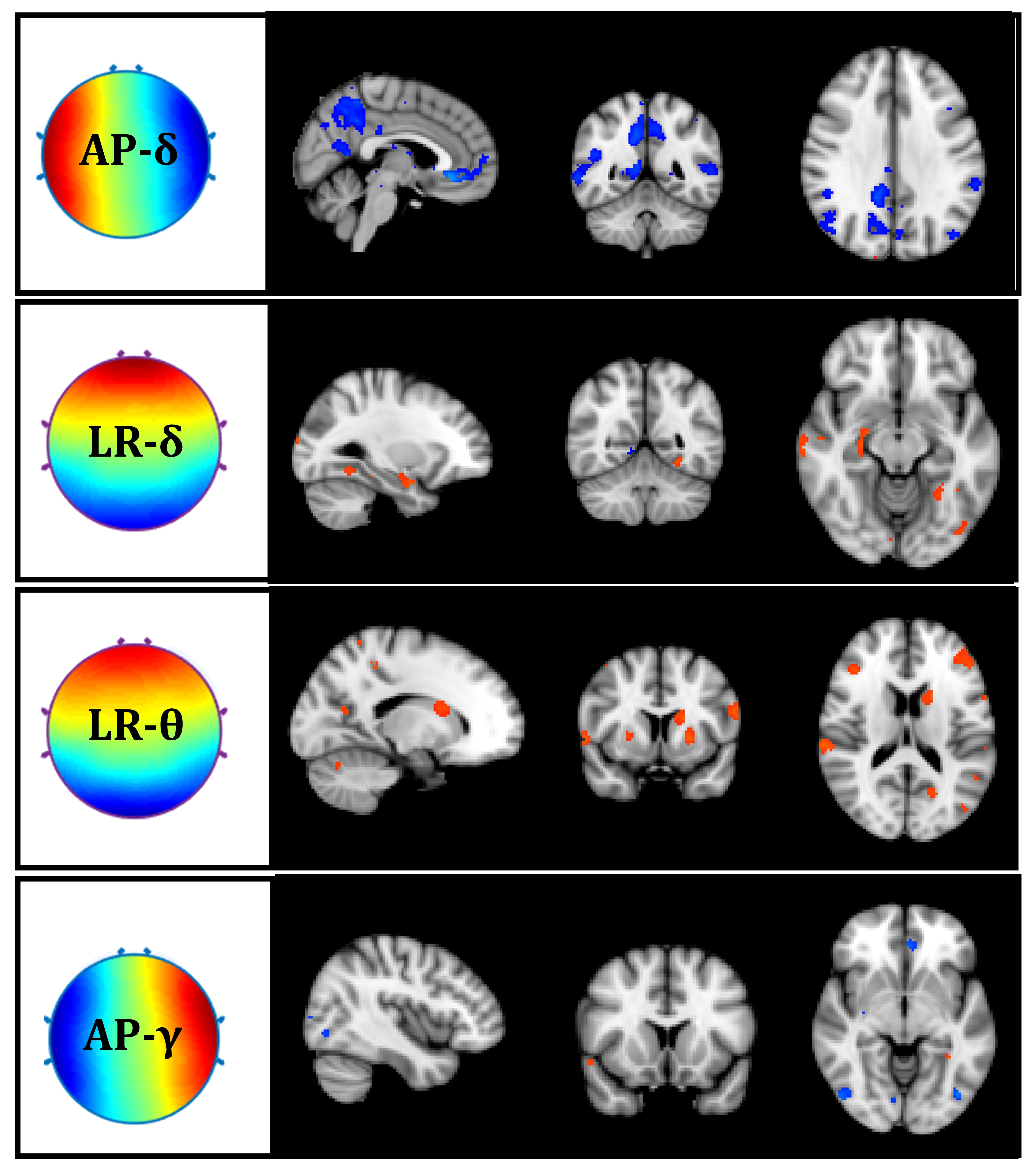


**Figure S1(b): Distractor-engagement**


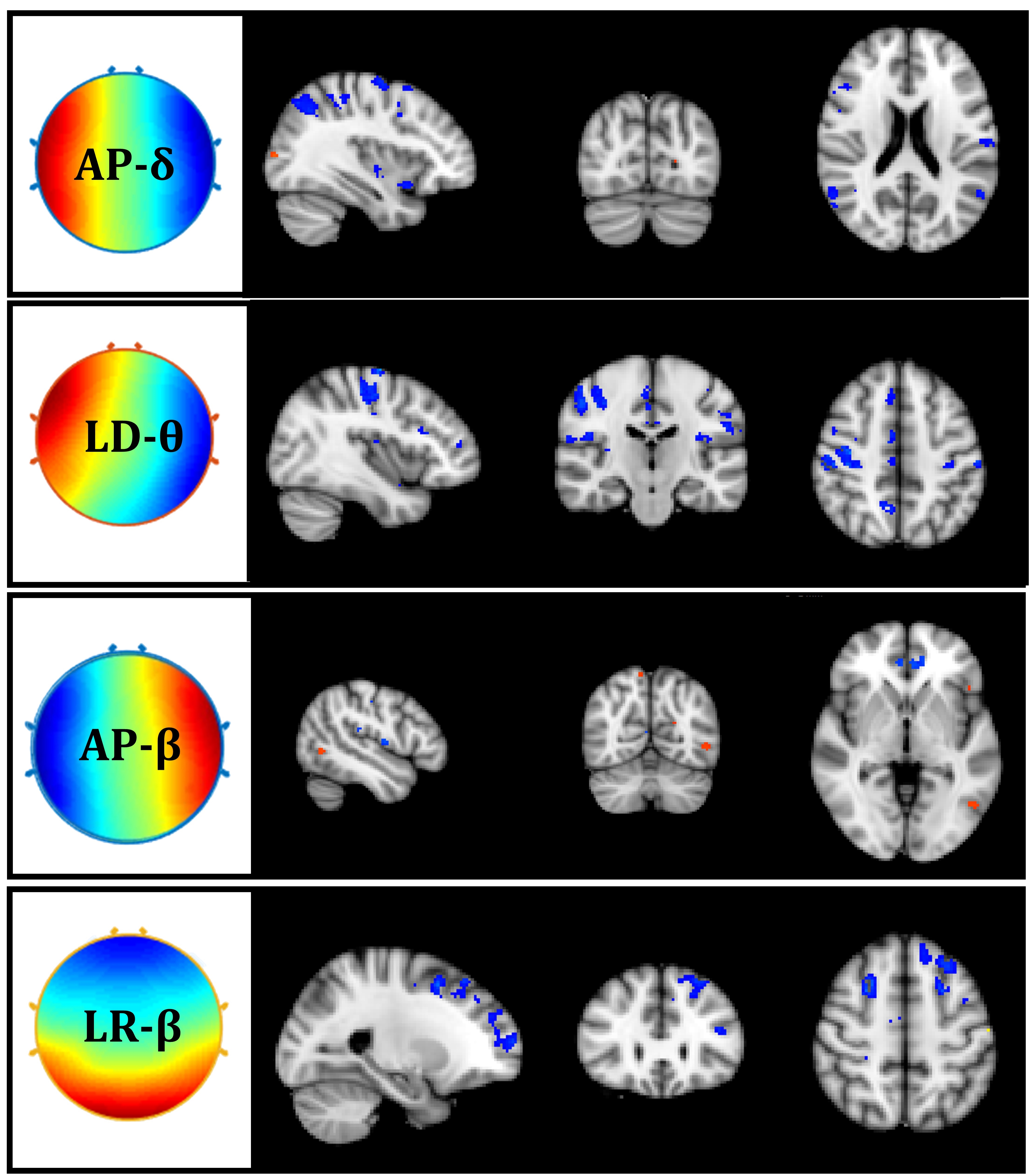


**Figure S1 (c): Fixation-engagement**

**Figure S1:** Neural correlates of frequency-microstates estimated using EEG informed fMRI analysis for a).Target-engagement b). Distractor-engagement and c). Fixation-engagement. The diagram represents the neural correlates only for those microstates, which are found significantly (p<0.01, FDR corrected) correlated with the neuronal engagement. Nomenclature for microstates are defined as AP (Anterior -Posterior), LR (Left-Right), LD (Left Diagonal), RD (Right Diagonal). The figures represent the statistically significant spatial maps localizing the clusters on the MNI brain with red clusters as positive and blue clusters as negative correlates of frequency-microstates.

**Additional file TABLES**:

**Table S1:** Neural correlates of task engagements ( target, distractor and fixation). C1 and C2 denote clusters at a significance level of (p<0.05). The details of cluster are depicted in a format of (Cluster:(x,y,z)/Laterality(L/R)/Cluster size/t-value). Laterality of neuronal activations has been represented as ‘R’ for the right hemisphere and ‘L’ for the left hemisphere.

| **Stimuli** | **Brain Region** | **Cluster information** |
| --- | --- | --- |
| **Target** | Frontal Orbital Cortex | C1:( -44.7,37.2,-4.65)/L/703/3.83;  C2:(-27.1,30.2,4.75)/L/11/2.37; |
|  | Frontal Pole | C1:( 45.5,36.9,-.52)/R/283/3.25; |
|  | Inferior Temporal Gyrus | C1:( -53,-49.8,-8.37)/L/394/3.11;  C2:(-52.2,-58.1,-20.2)/L/70/3.03; |
|  | Lateral Occipital Cortex | C1:( 10.8,-74.2,57)/R/21/2.48; |
|  | Middle Temporal Gyrus | C1:( 59.9,-33.5,-1.71)/R/192/3.39; |
|  | Occipital Pole | C1:( 1.39,-94.1,13)/R/150/3.56; |
|  | Paracingulate Gyrus | C1:( 6.85,35.3,34.5)/R/543/3.81;  C2:(-5.71,34.1,35.6)/L/509/3.63; |
|  | Precuneus Cortex | C1 :( 0.894,-71.7,59.5)/R/8/2.88; |
|  | Superior Frontal Gyrus | C1:( 17,34.7,47.3)/R/6/2.12; |
|  | Angular Gyrus | C1:(-50.1,-53.2,34.6)/L/836/3.74;  C2:(52.6,-49,42.7)/R/408/4.18;  C3:(46,-54.4,33.2)/R/5/2.47; |
|  | Insular Cortex | C1:(- 29,15.2,13.6)/L/10/2.1; |
|  | Temporal Occipital Fusiform Cortex | C1:( -33.5,-49.7,-1.11)/L/7/2.61; |
| **Distractor** | Frontal Pole | C1:( -18.9,60.6,14)/L/30/2.73;  C2:(-22.7,47.6,15.5)/L/22/2.58; |
|  | Angular Gyrus | C1:( -50.2,-58.5,30.9)/L/344/2.97; |
|  | Paracingulate Gyrus | C1:( -7.88,34.6,30.1)/L/375/3.26; |
|  | Temporal-Occipital Fusiform Cortex | C1:( 34.6,-42.2,-5.2)/R/20/2.76; |
|  | Intracalcarine Cortex | C1:( -21,-77,4.94)/L/37/2.69; |
|  | Occipital Fusiform Gyrus | C1:( -37,-66.5,-19.2)/L/1260/3.66; |
|  | Occipital Pole | C1:( 11.6,-90.6,15.1)/R/893/4.03; |
| **Fixation** | Inferior Frontal Gyrus | C1:(-39.5,25.7,19.9)/L/1614/3.69 |
|  | Intracalcarine Cortex | C1:(4.77,-83.6,8.4)/R/1515/4.19 |
|  | Lateral Occipital Cortex Superior | C1:(-28.6,-65.1,35)/L/1360/4.08  C2:(29,-57.7,53.2)/R/298/3.49; |
|  | Paracingulate Gyrus | C1:(-6.49,24.9,34.9)/L/1184;  C2:(7.92,26.1,34.8)/R/933; |
|  | Insular Cortex | C1:(-34.3,19,0)/L/817/4.67; |
|  | Precentral Gyrus | C1:(-29.6,-3.51,47.4)/L/54/2.84; |
|  | Lingual Gyrus | C1:(17.8,-67.9,-3.33)/R/32/2.98; |
|  | Lateral Occipital Cortex inferior | C1:(36.3,-77.3,4.46)/R/26/2.92; |
|  | Middle Frontal Gyrus | C1:(-34.6,0,55.7)/L/18/2.54; |
|  | Superior Frontal Gyrus | C1:(23.1,22.2,46.9)/R/2.93; |
|  | Superior Parietal Lobule | C1:(-19,-55.8,57)/L/12/2.21; |

**Table S2:** Summary table of the systematic literature review.

| **S.**  **N.** | **Study** | **Title** | **Modality used** | **Neural signature/ Method used** | **Task** | **Findings** |
| --- | --- | --- | --- | --- | --- | --- |
| 1 | **(Gerson et al., 2006)** | Cortically coupled computer vision for rapid image search | EEG | ERP | Visual Target detection (RSVP) | Single-trial EEG analysis is an efficient method for identifying the neural signatures. |
| 2 | **(Bigdely-Shamlo et al., 2008)** | Brain activity-based image classification from rapid serial visual presentation | EEG | ICA, Fisher LDA | Visual Target detection | Efficient for higher RSVP rate and smaller image size. |
| 3 | **(Wang & Jung, 2011)** | A Collaborative Brain-Computer Interface for Improving  Human Performance | EEG | ERP, Collaborative BCI | Delayed saccade task | Improved performance for greater user.  Faster estimation over motor response. |
| 4 | **(Brouwer et al., 2013)** | Distinguishing between target and nontarget fixations in a visual search task using fixation-related potentials | EEG, Eye tracking | ERP | Visual target detection | Target/nontarget FRPs can be distinguished on a single trial level. |
| 5 | **(Ganin et al., 2013)** | A P300-based Brain-Computer Interface with Stimuli on Moving Objects: Four-Session Single-Trial and Triple-Trial Tests with a Game-Like Task Design | EEG | ERP-P300, Fisher  discriminant analysis (LDA) | Visual oddball paradigm | The prospective for BCI games/robotics is due to little loss in classification accuracy. |
| 6 | **(Rohani & Puthusserypady, 2015)** | BCI inside a virtual reality classroom: a potential training tool for attention | EEG | ERP, Nonlinear optimized SVM | Visual oddball task | Low error rate. |
| 7 | **(Tang et al., 2016)** | A Brain-Machine Interface Based on ERD/ERS for an Upper-Limb Exoskeleton control | EEG | T-f analysis, LDA, SVM | Motor imagery | Greater classification accuracy for exoskeleton during online control phase. |
| 8 | **(Vareka & Mautner, 2017)** | Stacked Autoencoders for the P300 Component Detection | EEG | ERP, Stacked autoencoder | Visual target detection | Better accuracy of SAE classifier to LDA and MLP in P300 detection. |
| 9 | **(Pereira et al., 2017)** | EEG neural correlates of goal-directed movement intention | EEG | MRCP, sLDA | Reach-and-touch  task | Higher MRCP for goal at contralateral, central electrodes. |
| 10 | (Lin et al., 2017) | Multi rapid Serial Visual Presentation Framework for EEG-Based Target Detection | EEG | ERP | Visual target detection | Higher recognition accuracy for multi-RSVP. Improved accuracy for multiple target image. |
| 11 | **(Brouwer et al., 2017)** | EEG and Eye Tracking Signatures of Target Encoding during Structured Visual Search | EEG, Eye tracking | ERP-P300, Linear SVM | Visual target detection, Math task | Better EEG features using eye tracking to distinguish targets from non-targets. |
| 12 | **(Deshpande et al., 2017)** | A New Generation of Brain-Computer Interfaces Driven by Discovery of Latent EEG-fMRI Linkages Using Tensor Decomposition | EEG, fMRI | ERP-P300, SVM | Speller task | Latent linkages between electrical and hemodynamic signatures of neural activity, serve as a template. |
| 13 | **(Shojaedini et al., 2018)** | A new method for detecting P300 signals by using deep learning: Hyperparameter tuning in high-dimensional space by minimizing nonconvex error function | EEG | ERP-P300, CNN | Visual target detection | Minimizes nonconvex error function in high-dimensional space. |
| 14 | **(Zafar et al., 2018)** | A study of decoding human brain activities from simultaneous data of EEG and fMRI using MVPA | EEG, fMRI | Time-frequency analysis, MVPA, SVM, | Image classification task | EEG showed almost similar accuracy (65.7%) comparable to fMRI (64.16%) using MVPA, t-test and SVM. |
| 15 | **(Arvaneh et al., 2019)** | A P300-Based Brain-Computer Interface for Improving Attention | EEG | ERP-P300, T-f analysis, LDA | Random-dot motion, Speller task, | Enhanced ERP components, improvement in RT in the post-training. |
| 16 | **(Won et al., 2019)** | P300 Speller performance Predictor Based on RSVP Multi-feature | EEG | ERP-P300, multi-feature predictor | Speller task (RSVP) | higher predictability than any single feature predictor. |
| 17 | **(F. Li et al., 2020)** | A Novel P300 Classification Algorithm Based on a Principal Component Analysis-Convolutional Neural Network | EEG | ERP, PCA, CNN | Speller task | PCA-CNN with greater than 90% accuracy rate. |
| 18 | **(Nagabushanam et al., 2020)** | EEG signal classification using LSTM and improved neural network  algorithms | EEG | LSTM | Bonn database | Better performance accuracy with single stage LSTM. |
| 19 | **(Joshi et al., 2018)** | Single trial P300 classification using learning ensembles method convolutional LSTM and deep | EEG | ConvLSTM | Oddball task | ConvLSTM models the spatio-temporal information well. Better performance with less parameters. |
| 20 | **(Gong et al., 2020)** | An idle state-detecting method based on transient visual evoked potentials for an asynchronous ERP-based BCI | EEG | ERP-P300, VEP, Probability-based fisher LDA | Visual oddball paradigm | Improvement of P-FLDA over the FLDA due to ERP-VEP integration. |
| 21 | **(Zapała et al., 2020)** | The effects of handedness  on sensorimotor rhythm  desynchronization and  motor-imagery BCI control | EEG | T-f analysis, LDA | Motor imagery | Discrimination using  alpha/mu rhythms in SMR based BCI. |
| 22 | **(Mehdizavareh et al., 2020)** | Enhancing performance of subject-specific  models via subject-independent information  for SSVEP-based BCIs | EEG | CCA-based correlation coefficients | Cue-guided target selecting task. | Integration of subject-specific models with subject-independent information and enhances the BCI performance. |
| 23 | **(Vahid et al., 2020)** | Applying deep learning to single-trial EEG data provides evidence for complementary theories on action control | EEG | sLORETAEEGNet (LOOS approach) | Simon task for action control | Correct classification using markers from occipital/frontal areas |
| 24 | **(Kim & Kim, 2020)** | Identifying error features in a MI-BCI system using  microstates | EEG | EEG-Microstates | Motor imagery | Microstate MS5 showed a difference between correct and false responses. |
| 25 | **(DeLaRosa et al., 2020)** | Identification of selection and inhibition components in a Go/No-Go task from EEG spectra using a machine learning classifier | EEG | T-f analysis, single-layer neural network classifier | Go/No-Go task | Better assessment of time–frequency patterns using  neural network classifier (92%). |
| 26 | **(Huang et al., 2020)** | An Intelligent EEG Classification Methodology Based on Sparse Representation Enhanced Deep Learning Networks | EEG | Common spatial patterns (CSPs), CNN (FCR) | Motor Imagery | Better classification performance compared with sparse representation classification. |
| 27 | **(León et al., 2020)** | Deep learning for EEG-based Motor Imagery classification: Accuracy-cost trade-off | EEG | FFNN,CNN, RNN | Motor Imagery | Improved accuracy with hyperparameter optimization. Best results for CNN |
| 28 | **(Gao et al., 2020)** | Deep Convolutional Neural Network-Based Epileptic Electroencephalogram (EEG) Signal Classification | EEG | PSD energy diagrams (PSDEDs), Deep CNN | Epileptic EEG | Automatic feature extraction from PSDED, better classification with multichannel EEG signals. |
| 29 | **(Sikka et al., 2020)** | Investigating the temporal dynamics of electroencephalogram (EEG) microstates using recurrent neural networks | EEG | LSTM-based auto encoder (RNN) | Resting state | Microstates reconstruction by RNNs. |
| 30 | **(Sun et al., 2021)** | A hybrid deep neural network for classification of schizophrenia using EEG Data | EEG | T-f analysis, Hybrid DNN (CNN+LSTM) | Resting; Eyes open | DNN’s classification accuracy rate reached 99.22%. |
| 31 | **(Y. Li et al., 2021)** | A novel transferability attention neural network model for EEG emotion recognition | EEG | T-f analysis (STFT), Transferable attention neural N/w | Emotion recognition task | Improved recognition using TANN. At the sample level and brain-region-level. |

**References**

Abreu, R., Leal, A., & Figueiredo, P. (2018). EEG-informed fMRI: A review of data analysis methods. *Frontiers in Human Neuroscience*, *12*(February), 1–23. https://doi.org/10.3389/fnhum.2018.00029

Arvaneh, M., Robertson, I. H., & Ward, T. E. (2019). A P300-Based Brain-Computer Interface for Improving Attention. *Frontiers in Human Neuroscience*, *12*. https://doi.org/10.3389/fnhum.2018.00524

Behzadi, Y., Restom, K., Liau, J., & Liu, T. T. (2007). A component based noise correction method (CompCor) for BOLD and perfusion based fMRI. *NeuroImage*, *37*(1), 90–101. https://doi.org/10.1016/j.neuroimage.2007.04.042

Bigdely-Shamlo, N., Vankov, A., Ramirez, R. R., & Makeig, S. (2008). Brain activity-based image classification from rapid serial visual presentation. *IEEE Transactions on Neural Systems and Rehabilitation Engineering*, *16*(5), 432–441. https://doi.org/10.1109/TNSRE.2008.2003381

Brouwer, A. M., Hogervorst, M. A., Oudejans, B., Ries, A. J., & Touryan, J. (2017). EEG and eye tracking signatures of target encoding during structured visual search. *Frontiers in Human Neuroscience*, *11*(May), 1–11. https://doi.org/10.3389/fnhum.2017.00264

Brouwer, A. M., Reuderink, B., Vincent, J., van Gerven, M. A. J., & van Erp, J. B. F. (2013). Distinguishing between target and nontarget fixations in a visual search task using fixation-related potentials. *Journal of Vision*, *13*(3), 17. https://doi.org/10.1167/13.3.17

DeLaRosa, B. L., Spence, J. S., Motes, M. A., To, W., Vanneste, S., Kraut, M. A., & Hart, J. (2020). Identification of selection and inhibition components in a Go/NoGo task from EEG spectra using a machine learning classifier. *Brain and Behavior*, *10*(12), 1–15. https://doi.org/10.1002/brb3.1902

Deshpande, G., Rangaprakash, D., Oeding, L., Cichocki, A., & Hu, X. P. (2017). A new generation of brain-computer interfaces driven by discovery of latent EEG-fMRI linkages using tensor decomposition. *Frontiers in Neuroscience*, *11*(JUN), 1–13. https://doi.org/10.3389/fnins.2017.00246

Ganin, I. P., Shishkin, S. L., & Kaplan, A. Y. (2013). A P300-based brain-computer interface with stimuli on moving objects: Four-session single-trial and triple- trial tests with a game-like task design. *PLoS ONE*, *8*(10), 1–21. https://doi.org/10.1371/journal.pone.0077755

Gao, Y., Gao, B., Chen, Q., Liu, J., & Zhang, Y. (2020). Deep convolutional neural network-based epileptic electroencephalogram (EEG) signal classification. *Frontiers in Neurology*, *11*(May), 1–11. https://doi.org/10.3389/fneur.2020.00375

Gerson, A. D., Parra, L. C., & Sajda, P. (2006). Cortically coupled computer vision for rapid image search. *IEEE Transactions on Neural Systems and Rehabilitation Engineering*, *14*(2), 174–179. https://doi.org/10.1109/TNSRE.2006.875550

Gong, M., Xu, G., Li, M., & Lin, F. (2020). An idle state-detecting method based on transient visual evoked potentials for an asynchronous ERP-based BCI. *Journal of Neuroscience Methods*, *337*(March), 108670. https://doi.org/10.1016/j.jneumeth.2020.108670

Huang, J. S., Li, Y., Chen, B. Q., Lin, C., & Yao, B. (2020). An Intelligent EEG Classification Methodology Based on Sparse Representation Enhanced Deep Learning Networks. *Frontiers in Neuroscience*, *14*(September), 1–8. https://doi.org/10.3389/fnins.2020.00808

Huster, R. J., Debener, S., Eichele, T., & Herrmann, C. S. (2012). Methods for simultaneous EEG-fMRI: An introductory review. *Journal of Neuroscience*, *32*(18), 6053–6060. https://doi.org/10.1523/JNEUROSCI.0447-12.2012

Joshi, R., Goel, P., Sur, M., & Murthy, H. A. (2018). Single trial P300 classification using convolutional LSTM and deep learning ensembles method. In *Lecture Notes in Computer Science (including subseries Lecture Notes in Artificial Intelligence and Lecture Notes in Bioinformatics): Vol. 11278 LNCS*. Springer International Publishing. https://doi.org/10.1007/978-3-030-04021-5_1

Kim, S. K., & Kim, L. (2020). Identifying error features in a MI-BCI system using microstates. *8th International Winter Conference on Brain-Computer Interface, BCI 2020*, 1–3. https://doi.org/10.1109/BCI48061.2020.9061613

León, J., Escobar, J. J., Ortiz, A., Ortega, J., González, J., Martín-Smith, P., Gan, J. Q., & Damas, M. (2020). Deep learning for EEG-based motor imagery classification: Accuracy-cost trade-off. *PLoS ONE*, *15*(6), 1–30. https://doi.org/10.1371/journal.pone.0234178

Li, F., Li, X., Wang, F., Zhang, D., Xia, Y., & He, F. (2020). A novel P300 classification algorithm based on a principal component analysis-convolutional neural network. *Applied Sciences (Switzerland)*, *10*(4), 1–15. https://doi.org/10.3390/app10041546

Li, Y., Fu, B., Li, F., Shi, G., & Zheng, W. (2021). A novel transferability attention neural network model for EEG emotion recognition. *Neurocomputing*, *447*, 92–101. https://doi.org/10.1016/j.neucom.2021.02.048

Lin, Z., Zeng, Y., Wang, X., Wu, Q., & Yan, B. (2017). Multirapid Serial Visual Presentation Framework for EEG-Based Target Detection. *International IEEE/EMBS Conference on Neural Engineering, NER*, *2017*, 556–559. https://doi.org/10.1109/NER.2017.8008412

Mehdizavareh, M. H., Hemati, S., & Soltanian-Zadeh, H. (2020). Enhancing performance of subject-specific models via subject-independent information for SSVEP-based BCIs. *PLoS ONE*, *15*(1), 1–20. https://doi.org/10.1371/journal.pone.0226048

Murray, M. M., Brunet, D., & Michel, C. M. (2008). Topographic ERP analyses: A step-by-step tutorial review. *Brain Topography*, *20*(4), 249–264. https://doi.org/10.1007/s10548-008-0054-5

Nagabushanam, P., Thomas George, S., & Radha, S. (2020). EEG signal classification using LSTM and improved neural network algorithms. *Soft Computing*, *24*(13), 9981–10003. https://doi.org/10.1007/s00500-019-04515-0

Pascual-Marqui, R. D., Michel, C. M., & Lehmann, D. (1995). Segmentation of Brain Electrical Activity into Microstates; Model Estimation and Validation. *IEEE Transactions on Biomedical Engineering*, *42*(7), 658–665. https://doi.org/10.1109/10.391164

Pereira, J., Ofner, P., Schwarz, A., Sburlea, A. I., & Müller-Putz, G. R. (2017). EEG neural correlates of goal-directed movement intention. *NeuroImage*, *149*(January), 129–140. https://doi.org/10.1016/j.neuroimage.2017.01.030

Pernet, C. R., Wilcox, R., & Rousselet, G. A. (2013). Robust correlation analyses: False positive and power validation using a new open source matlab toolbox. *Frontiers in Psychology*, *3*(JAN), 1–18. https://doi.org/10.3389/fpsyg.2012.00606

Rohani, D. A., & Puthusserypady, S. (2015). BCI inside a virtual reality classroom: a potential training tool for attention. *EPJ Nonlinear Biomedical Physics*, *3*(1). https://doi.org/10.1140/epjnbp/s40366-015-0027-z

Sikka, A., Jamalabadi, H., Krylova, M., Alizadeh, S., van der Meer, J. N., Danyeli, L., Deliano, M., Vicheva, P., Hahn, T., Koenig, T., Bathula, D. R., & Walter, M. (2020). Investigating the temporal dynamics of electroencephalogram (EEG) microstates using recurrent neural networks. *Human Brain Mapping*, *41*(9), 2334–2346. https://doi.org/10.1002/hbm.24949

Sun, J., Cao, R., Zhou, M., Hussain, W., Wang, B., Xue, J., & Xiang, J. (2021). A hybrid deep neural network for classification of schizophrenia using EEG Data. *Scientific Reports*, *11*(1), 1–16. https://doi.org/10.1038/s41598-021-83350-6

Tang, Z., Sun, S., Zhang, S., Chen, Y., Li, C., & Chen, S. (2016). A brain-machine interface based on ERD/ERS for an upper-limb exoskeleton control. *Sensors (Switzerland)*, *16*(12), 1–14. https://doi.org/10.3390/s16122050

Vahid, A., Mückschel, M., Stober, S., Stock, A. K., & Beste, C. (2020). Applying deep learning to single-trial EEG data provides evidence for complementary theories on action control. *Communications Biology*, *3*(1). https://doi.org/10.1038/s42003-020-0846-z

Vareka, L., & Mautner, P. (2017). Stacked autoencoders for the P300 component detection. *Frontiers in Neuroscience*, *11*(MAY). https://doi.org/10.3389/fnins.2017.00302

Vaswani, A., et al. (2017). Attention Is All You Need. *IEEE Industry Applications Magazine*, *8*(1), 8–15. https://doi.org/10.1109/2943.974352

Wager, T. D., Keller, M. C., Lacey, S. C., & Jonides, J. (2005). Increased sensitivity in neuroimaging analyses using robust regression. *NeuroImage*, *26*(1), 99–113. https://doi.org/10.1016/j.neuroimage.2005.01.011

Wang, Y., & Jung, T. P. (2011). A collaborative brain-computer interface for improving human performance. *PLoS ONE*, *6*(5). https://doi.org/10.1371/journal.pone.0020422

Whitfield-gabrieli, S., & Nieto-castanon, A. (2012). *Conn : A Functional Connectivity Toolbox for Correlated and Anticorrelated Brain Networks*. *2*(3). https://doi.org/10.1089/brain.2012.0073

Won, K., Kwon, M., Jang, S., Ahn, M., & Jun, S. C. (2019). P300 Speller Performance Predictor Based on RSVP Multi-feature. *Frontiers in Human Neuroscience*, *13*. https://doi.org/10.3389/fnhum.2019.00261

Zafar, R., Kamel, N., Naufal, M., Saeed, A., Sarat, M., Rana, C. D., & Ahmad, F. (2018). A study of decoding human brain activities from simultaneous data of EEG and fMRI using MVPA. *Australasian Physical & Engineering Sciences in Medicine*, *0*(0), 0. https://doi.org/10.1007/s13246-018-0656-5

Zapała, D., Zabielska-Mendyk, E., Augustynowicz, P., Cudo, A., Jaśkiewicz, M., Szewczyk, M., Kopiś, N., & Francuz, P. (2020). The effects of handedness on sensorimotor rhythm desynchronization and motor-imagery BCI control. *Scientific Reports*, *10*(1), 1–11. https://doi.org/10.1038/s41598-020-59222-w

Zhang, G., Davoodnia, V., Sepas-Moghaddam, A., Zhang, Y., & Etemad, A. (2020). Classification of Hand Movements from EEG Using a Deep Attention-Based LSTM Network. *IEEE Sensors Journal*, *20*(6), 3113–3122. https://doi.org/10.1109/JSEN.2019.2956998
